# Differential repression of *Otx2* underlies the capacity of NANOG and ESRRB to induce germline entry

**DOI:** 10.1016/j.stemcr.2021.11.013

**Published:** 2021-12-30

**Authors:** Matúš Vojtek, Jingchao Zhang, Juanjuan Sun, Man Zhang, Ian Chambers

**Affiliations:** 1 Centre for Regenerative Medicine, Institute for Stem Cell Research, School of Biological Sciences, University of Edinburgh, 5 Little France Drive, Edinburgh EH16 4UU, Scotland; 2 Center for Cell Lineage and Atlas (CCLA), Bioland Laboratory, Guangzhou Regenerative Medicine and Health Guangdong Laboratory, Guangzhou, China; 3 The Fifth Affiliated Hospital of Guangzhou Medical University, Guangzhou, China; 4 Guangzhou Laboratory, No. 9 XingDaoHuanBei Road, Guangzhou International Bio Island, Guangzhou, 510005, Guangdong Province, China

**Keywords:** embryonic stem cells, germline, primordial germ cells, epiblast-like cells, Nanog, Esrrb, Otx2

## Abstract

Primordial germ cells (PGCs) arise from cells of the post-implantation epiblast in response to cytokine signaling. PGC development can be recapitulated *in vitro* by differentiating epiblast-like cells (EpiLCs) into PGC-like cells (PGCLCs) through cytokine exposure. Interestingly, the cytokine requirement for PGCLC induction can be bypassed by enforced expression of the transcription factor (TF) NANOG. However, the underlying mechanisms are not fully elucidated. Here, we show that NANOG mediates *Otx2* downregulation in the absence of cytokines and that this is essential for PGCLC induction by NANOG. Moreover, the direct NANOG target gene *Esrrb*, which can substitute for several NANOG functions, does not downregulate *Otx2* when overexpressed in EpiLCs and cannot promote PGCLC specification*.* However, expression of ESRRB in *Otx2*^+/−^ EpiLCs rescues emergence of PGCLCs. This study illuminates the interplay of TFs occurring at the earliest stages of PGC specification.

## Highlights

• NANOG overexpression induces cytokine-free PGCLC specification by repressing *Otx2*
• Enforced OTX2 expression prevents NANOG-induced germline entry
• ESRRB overexpression cannot repress *Otx2* or induce cytokine-free germline entry
• *Otx2* heterozygosity enables ESRRB to induce cytokine-free PGCLC specification

## Teaser

Vojtek and colleagues show that NANOG represses *Otx2* to induce cytokine-free specification of primordial germ cell-like cells (PGCLCs). OTX2 levels are critical for PGCLC induction, as sustained OTX2 expression prevents NANOG-induced differentiation. Although ESRRB can functionally substitute for NANOG in other processes, ESRRB does not repress *Otx2* and cannot induce cytokine-free PGCLC specification unless the *Otx2* expression is halved.

## Introduction

Germline development and sexual reproduction depend on the establishment of primordial germ cells (PGCs). In mice, PGCs are induced mainly by bone morphogenic factor 4 (BMP4) and other cytokines, including BMP8a and BMP2, that act on proximal posterior epiblast cells at embryonic day 6 (Hayashi et al., 2007; Lawson et al., 1999). Prospective PGCs downregulate the epiblast transcription factor (TF) OTX2 and subsequently activate the key PGC TFs BLIMP1 (PRDM1), PRDM14 and AP2γ (Kurimoto et al., 2008; Ohinata et al., 2005; Vincent et al., 2005; Weber et al., 2010; Yamaji et al., 2008; Zhang and Chambers, 2019). These events can be recapitulated *in vitro* by differentiating naive embryonic stem cells (ESCs) into epiblast-like cells (EpiLCs), which are transiently competent to specify PGC-like cells (PGCLCs) in response to BMP4 and associated cytokines (Hayashi et al., 2011; Hayashi and Saitou, 2013). The requirement for BMP4 and associated cytokines can however be bypassed by induction of NANOG in EpiLCs (Murakami et al., 2016). Combined ectopic expression of NANOG and cytokine treatment further expands the proportion of PGCLCs in the differentiated population (Murakami et al., 2016). Even though *Nanog* is not essential for the emergence of PGCs, or for germline transmission (Carter et al., 2014; Zhang et al., 2018b), *Nanog* deletion results in a large decrease in PGC numbers both *in vitro* and *in vivo* (Chambers et al., 2007; Murakami et al., 2016; Yamaguchi et al., 2009; Zhang et al., 2018b).

The target genes through which NANOG acts in ESCs have been identified and include *Esrrb* and *Otx2*, which are regulated positively and negatively by NANOG, respectively (Festuccia et al., 2012). Both of these genes also regulate the PGC compartment (Mitsunaga et al., 2004; Zhang et al., 2018a). Germline loss of function for *Esrrb* results in a similar quantitative reduction in PGC numbers at mid-gestation, as seen when *Nanog* is deleted specifically from the germline (Mitsunaga et al., 2004; Zhang et al., 2018b). Moreover, deletion of *Nanog* impairs induction of PGCLC differentiation in response to PGC-promoting cytokines (Murakami et al., 2016). This absence of PGC differentiation can be compensated for by enforced expression of ESRRB (Zhang et al., 2018b). Consistent with a conserved epistatic relationship between Nanog and Esrrb both in the preimplantation epiblast and in the germline, knockin of *Esrrb* to the *Nanog* locus overcomes the reduction in PGC numbers resulting from germline-specific *Nanog* deletion (Zhang et al., 2018b).

In ESCs, Otx2 and Nanog antagonize each other by mutual repression (Acampora et al., 2017; Festuccia et al., 2012). We have shown that entry to the germline is blocked when OTX2 expression is maintained during the first 2 days of EpiLC-PGCLC differentiation (Zhang et al., 2018a; Zhang and Chambers, 2019). In contrast, *Otx2* deletion dramatically increases PGCLC numbers *in vitro* and raises PGC numbers *in vivo* (Zhang et al., 2018a). Recently, OTX2 has been shown to act through *cis*-acting binding sites that repress transcription of *Nanog* and *Pou5f1* (*Oct4*) (Di Giovannantonio et al., 2021). However, despite increasing knowledge of the relationships among ESRRB, NANOG, and OTX2, the interplay among these factors in PGC induction is not fully understood. Here we assess the capacity of ESRRB and NANOG to induce PGCLC differentiation using inducible transgene systems in the absence of cytokines. Our results uncover a differential capacity of NANOG and ESRRB to repress *Otx2* and an OTX2 dose-dependent barrier to germline induction by ESRRB and NANOG in the absence of cytokines.

## Results

### ESRRB cannot activate the PGCLC program

**Figure 1:**
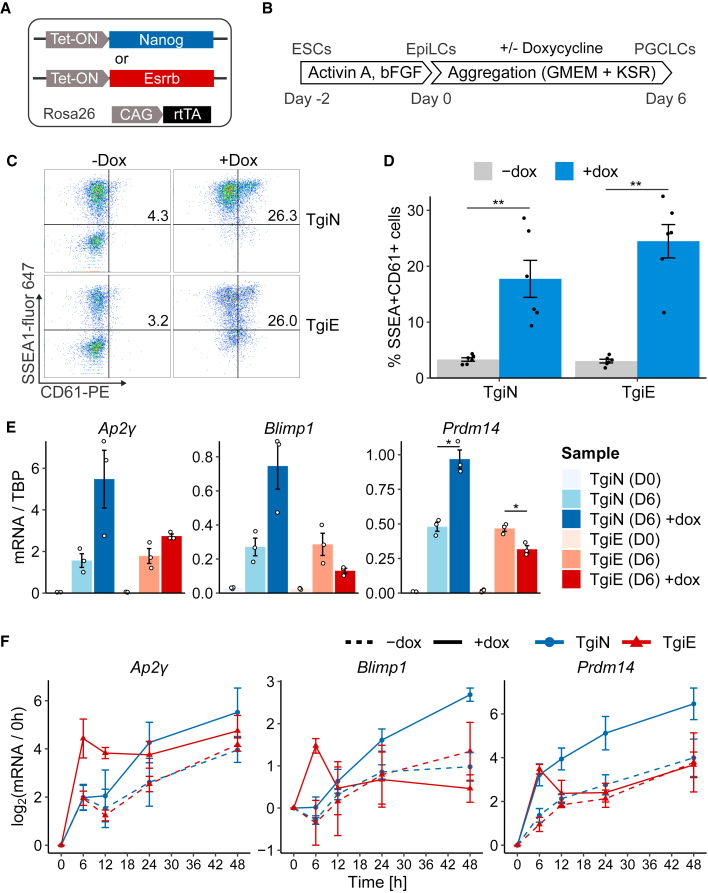
ESRRB cannot activate the PGC program (A) Cartoon of TetON-Nanog (TgiN) and TetON-Esrrb (TgiE) cell lines. TetON expression cassettes were randomly integrated into E14Tg2a cells expressing rtTA from *Rosa26*. (B) The cytokine-free PGCLC differentiation protocol. ESCs cultured in 2i/LIF were differentiated to EpiLCs by culture in Activin A and bFGF for 2 days. EpiLCs were then aggregated in GMEM+KSR medium for 6 days with (+dox) or without (−dox) doxycycline, and PGCLC status was analyzed. (C) Representative example of flow cytometry analysis of PGCLC aggregates from TgiN and TgiE cells at day 6 with or without dox, showing the percentage of SSEA1^+^CD61^+^ cells. (D) Quantification of (C). Bars are mean ± SEM, points are individual data measurements (n = 6 independent experiments). (E) qRT-PCR of indicated transcripts in TgiN and TgiE cells at day 6 of cytokine-free PGCLC differentiation with or without dox. Bars represent mean mRNA levels normalized to *Tbp* mRNA. Bars are mean ± SEM, and points are individual data measurements (n = 3 independent experiments). (F) Time-course analysis of the indicated mRNAs during the first 48 h of cytokine-independent PGCLC differentiation of TgiN and TgiE EpiLCs with or without dox. Points, triangles, and lines represent the mean log_2_ fold change in ratio between *Tbp*-normalized expression and the zero time point (mean ± SD; n = 3 independent experiments). ^∗^p < 0.05 and ^∗∗^p < 0.01 (t test).

To examine whether ESRRB can induce cytokine-free PGCLC differentiation similarly to NANOG, we overexpressed NANOG or ESRRB during cytokine-free PGCLC differentiation. Cell lines carrying tetracycline-inducible Nanog (TgiN) or Esrrb (TgiE) transgenes were generated by integrating piggyBac transposons into E14Tg2a ESCs expressing the modified reverse tetracycline transactivator (rtTA2) (Urlinger et al., 2000) from *Rosa26* (Figure 1A). The resulting TgiN and TgiE ESCs were then differentiated to PGC-competent epiblast-like cells (Figure 1B). In the original PGCLC differentiation protocol, EpiLCs are aggregated in a cytokine cocktail that is required for PGCLC specification (Hayashi et al., 2011; Hayashi and Saitou, 2013). However, NANOG can direct PGCLC differentiation in the absence of cytokines (Murakami et al., 2016). We therefore omitted cytokines and tested the ability of NANOG or ESRRB overexpression to induce PGCLC development (Figure 1B). TgiN and TgiE cells formed similar colonies both as naive ESCs and EpiLCs (Figure S1A). Doxycycline addition during PGCLC specification from EpiLCs activated robust induction of Nanog or Esrrb transgenes in TgiN or TgiE cells, respectively (Figure S1B). Induced expression of either *Nanog* or *Esrrb* resulted in similar levels of surface expression of SSEA1 and CD61, which jointly mark PGCLCs (Hayashi et al., 2011) (Figures 1C, 1D, and S2A). However, in contrast to NANOG, induction of ESRRB failed to increase expression of *Blimp1* or *Prdm14* mRNAs and showed only a modest increase in *Ap2γ* mRNA (Figure 1E). We therefore assessed changes in PGC TF expression earlier, during the first 48 h of differentiation (Figures 1F and S1B). Induction of NANOG activated expression of both *Esrrb* and the PGC transcription factors *Blimp1*, *Prdm14*, and *Ap2γ*. Interestingly, while ESRRB induction increased *Blimp1*, *Prdm14*, and *Ap2γ* mRNAs within the first 6 h of differentiation, ESRRB did not sustain *Blimp1* and *Prdm14* expression at later times (Figure 1F). Therefore, ESRRB unlike NANOG does not produce a sustained activation of the PGC program during cytokine-free PGCLC differentiation.

### NANOG induces PGCLCs by repressing *Otx2*

**Figure 2:**
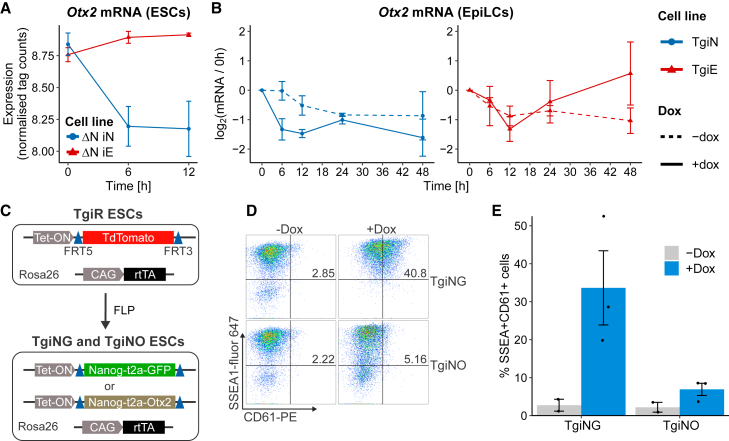
*Otx2* downregulation is essential for PGCLC induction by NANOG (A) Relative changes of *Otx2* mRNA in *Nanog*^−/−^ TetON-Nanog (ΔN-iN) or *Nanog*^−/−^ TetON-Esrrb (ΔN-iE) ESCs at indicated time points after doxycycline treatment (mean ± SD, n = 3 independent experiments). Data adapted from Festuccia et al. (2012). (B) Relative changes of *Otx2* mRNA after aggregation of TgiN and TgiE EpiLCs cultured with (+dox) or without (−dox) doxycycline. Lines, points, and triangles represent the mean log_2_ in ratio between *Tbp*-normalized expression and the zero time point (mean ± SD; n = 3 independent experiments). (C) *Rosa26*: rtTA; E14Tg2a-TetON-TdTomato (TgiR) ESCs were modified as shown to derive inducible Nanog-t2a-GFP (TgiNG) or Nanog-t2a-Otx2 (TgiNO) ESCs. (D) Representative example of flow cytometry analysis of PGCLC aggregates from TgiNG and TgiNO cells at day 6 with or without dox. Percentages of SSEA1^+^CD61^+^ cells are shown. (E) Quantification of (D). Bars are mean ± SEM, points are individual data measurements (n = 2 independent experiments for −dox, and n = 3 for +dox).

Our previous results show that the requirement of cytokines for PGCLC formation is also eliminated in *Otx2*^−/−^ cells (Zhang et al., 2018a). OTX2 and NANOG have antagonistic functions in ESCs (Acampora et al., 2017). Moreover, NANOG can directly downregulate *Otx2* in ESCs (Figure 2A; Festuccia et al., 2012; Heurtier et al., 2019). Microarray data suggest that this capacity to repress *Otx2* may not be shared by ESRRB (Figure 2A; Festuccia et al., 2012). This raises the hypothesis that ESRRB cannot effectively induce PGCLC specification because of an impaired capacity to repress *Otx2*. To address this, we quantified *Otx2* mRNA during the first 48 h of EpiLC aggregation of TgiN and TgiE cells in cytokine-free medium (Figure 1B). As expected, NANOG induction drove a rapid decrease in *Otx2* between 6 and 12 h compared with uninduced cells (Figure 2B). In contrast, ESRRB induction did not affect *Otx2* mRNA levels during the first 24 h (Figure 2B).

We next tested whether OTX2 clearance is necessary for NANOG to induce PGCLCs. To do this, we generated E14Tg2a ESC lines that can induce either GFP (TgiNG) or OTX2 (TgiNO) from the same transgene that induces NANOG (Figure 2C). TgiNG and TgiNO cells were generated by replacing TdTomato in E14Tg2a TetON-TdTomato (TgiR) cells with cassettes encoding Nanog-t2a-GFP or Nanog-t2a-Otx2 (Figure 2C). Doxycycline treatment increased *Nanog* expression ∼8- and 5-fold in TgiNG and TgiNO cells, respectively (Figure S2B). In addition, *Otx2* mRNA was induced by doxycycline only in TgiNO cells (Figure S2B). To assess the effect of OTX2 on the ability of NANOG to induce PGCLC differentiation, TgiNG and TgiNO cells were subjected to PGCLC differentiation in the absence of cytokines. Simultaneous induction of GFP and NANOG upregulated expression of PGCLC surface markers CD61 and SSEA1 (Figures 2D and 2E). In contrast, simultaneous induction of OTX2 and NANOG markedly reduced the population of SSEA1^+^/CD61^+^ cells (Figures 2D and 2E). This indicates that the capacity of NANOG to function in PGCLC induction requires repression of *Otx2*.

### *Otx2* heterozygosity enables ESRRB to induce cytokine-free PGC differentiation

**Figure 3:**
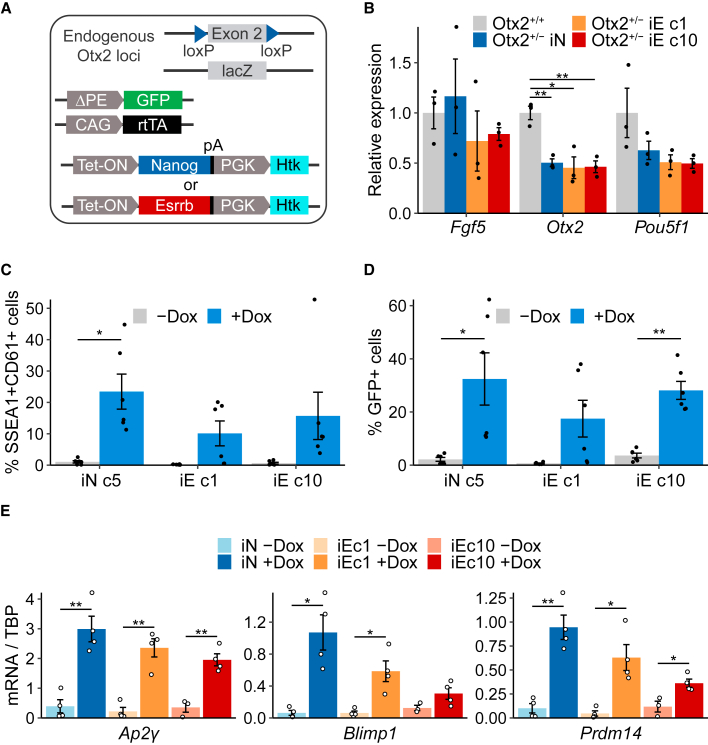
A reduced *Otx2* gene dose facilitates cytokine-free germline entry by ESRRB induction (A) Diagram of *Otx2*^+/−^: *Oct4* ΔPE-GFP ESCs carrying tetracycline-inducible Nanog (iN) or Esrrb (iE) transgenes. (B) Relative expression levels of indicated mRNAs in *Otx2*^+/−^ iN and iE (c1 and c10) EpiLCs in the absence of doxycycline. *Tbp*-normalized expression values are shown relative to the mean of *Tbp*-normalized expression of *Otx2*^*+/+*^ samples. Bars are mean ± SEM and points are individual data measurements (n = 3 independent experiments). (C) Size of PGCLC population (represented by SSEA1^+^CD61^+^ cells) in the presence (+dox) or absence (−dox) of doxycycline at day 6 of cytokine-free PGCLC differentiation of the indicated *Otx2*^+/−^ EpiLCs. Bars are mean ± SEM, and points are individual data measurements (n = 6 independent experiments). (D) Proportion of *Oct4* ΔPE-GFP^+^ cells in the indicated *Otx2*^*+/−*^ aggregates at day 6 of the cytokine-free PGCLC differentiation. Bars represent mean ± SEM of percentage of GFP+ cells, n = 6 independent experiments; points are individual data measurements. (E) qRT-PCR analysis of the indicated mRNAs relative to *Tbp* expression in day 6 aggregates from (C). Bars are mean ± SEM and points are individual data measurements (n = 4 independent experiments). ^∗^p < 0.05 and ^∗∗^p < 0.01 (t test).

To test whether OTX2 is a limiting factor that prevents ESRRB from activating PGCLC specification, we integrated doxycycline-inducible Nanog (iN) or Esrrb (iE) transgenes into heterozygous *Otx2*^lacZ/fl^ ESCs (Acampora et al., 2013; Zhang et al., 2018a) (Figure 3A). This cell line also contains a GFP transgene that reports the activity of the *Oct4* distal enhancer (ΔPE::GFP) (Figure 3A) which becomes activated in PGCLCs (Magnusdottir et al., 2013). We refer to these cells as *Otx2*^+/−^ iE and *Otx2*^+/−^ iN cells. We isolated two *Otx2*^+/−^ iE clones (1 and 10) and one *Otx2*^+/−^ iN clone that each express approximately 50% the level of *Otx2* mRNA compared with *Otx2*^+/+^ cells in the EpiLC state (Figure 3B).

Doxycycline treatment of *Otx2*^+/−^ iN and *Otx2*^+/−^ iE cell lines increased expression of the transgenes by 20- to 40-fold compared with wild-type cells at day 2 of cytokine-free PGCLC differentiation (Figure S3A). When *Otx2*^+/−^ cell lines were subjected to cytokine-free PGCLC induction in the absence of doxycycline, surface expression of CD61/SSEA1 was not induced in iE or iN cell lines (Figure S3B). However, upon induction of either NANOG or ESRRB by doxycycline, surface expression of CD61/SSEA1 was induced (Figures 3C and S3B). In addition, the proportion of cells expressing the Oct4 ΔPE::GFP transgene was similarly induced in each of these lines by doxycycline (Figure 3D). Furthermore, induction of either ESRRB or NANOG in *Otx2*^*+/−*^ cells increased expression of *Blimp1*, *Prdm14*, and *Ap2γ* mRNAs at day 6 (Figure 3E). This contrasts with induction of ESRRB in *Otx2*^*+/+*^ cells, which did not increase *Blimp1* and *Prdm14* mRNA levels (Figure 1E). Interestingly, at the earlier time point of day 2 of differentiation, ESRRB induced lower levels of *Blimp1*, *Prdm14*, and *Ap2γ* mRNA expression in *Otx2*^*+/−*^ cells than were achieved by NANOG induction (Figure S3A). This may indicate that ESRRB-induced germline entry in *Otx2*^*+/−*^ cells is delayed compared with that induced by NANOG.

## Discussion

A key function of PGC-specifying cytokines during the early stages of EpiLC-PGCLC differentiation is to rapidly downregulate *Otx2* (Zhang et al., 2018a). The results reported here show that NANOG induction in EpiLCs downregulates *Otx2* early during differentiation without requiring PGC-promoting cytokines. Importantly, these cytokines are also unnecessary for enforced NANOG expression in EpiLCs to induce PGCLCs (Murakami et al., 2016). The importance of the NANOG-mediated repression of *Otx2* for PGCLC differentiation under these conditions is shown by the reduction in the size of the PGCLC population when NANOG and OTX2 are simultaneously induced. Therefore, OTX2 depletion is essential for PGCLC specification by NANOG in the absence of cytokines.

ESRRB can substitute for NANOG in LIF-independent maintenance of mouse ESCs and in reprogramming of cells to naive pluripotency (Festuccia et al., 2012). In addition, knockin of *Esrrb* to *Nanog* rescues the reduced PGC numbers seen when *Nanog* is specifically deleted from the germ cell lineage (Zhang et al., 2018b). Furthermore, the failure of *Nanog*^−/−^ cells to maintain a PGCLC population in the presence of PGC-promoting cytokines is rescued by enforced ESRRB expression (Zhang et al., 2018b). It was therefore surprising to see that ESRRB, unlike NANOG, cannot efficiently induce PGC transcription factors *Blimp1* and *Prdm14* in the absence of cytokines. However, ESRRB overexpression in *Nanog*^*−/−*^ ESCs does not fully recapitulate the effect of NANOG overexpression on ESC self-renewal (Festuccia et al., 2012). Although NANOG and ESRRB share common target genes (Festuccia et al., 2012), distinct subsets of genes are regulated by either TF in ESCs (Sevilla et al., 2021). In addition, although the interactomes of NANOG and ESRRB contain common binding partners (Gagliardi et al., 2013; van den Berg et al., 2010), ESRRB, but not NANOG, interacts with Mediator, suggesting that ESRRB may function prominently in transcriptional initiation. Although both interactomes link to NuRD and PcG, NANOG also links to Sin3a and NcoR complexes. Therefore, NANOG may link more to transcriptional repression than ESRRB. These distinctions are consistent with the prominent role proposed for transcriptional repression during PGCLC differentiation (Kurimoto et al., 2008). Consistent with this, we show here that the failure of enforced ESRRB expression to recapitulate the PGCLC differentiation induced by ectopic NANOG may be due to the inability of ESRRB to rapidly downregulate *Otx2* within the competence time window required to initiate PGC differentiation. When we tested the effect of ESRRB induction in *Otx2*^*+/−*^ EpiLCs, ESRRB successfully activated expression of *Blimp1*, *Prdm14*, *Ap2γ*, and *Oct4* distal enhancer activity and surface expression of CD61/SSEA1. Therefore, the inability of ESRRB to induce the germline program is overcome by reducing the OTX2 level in the starting population.

**Figure 4:**
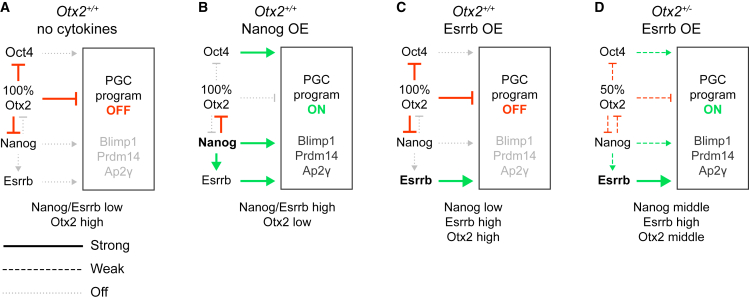
Proposed model of PGC induction (A) In wild-type EpiLCs, in the absence of BMP and associated cytokines, OTX2 levels remain above the threshold level required to block PGCLC differentiation during the period of germline competence. (B) If NANOG is overexpressed (OE) in EpiLCs at the start of differentiation, *Otx2* is repressed, leading to a decreased OTX2 level that no longer represses expression of the OTX2 target genes *Nanog* and *Oct4* (Di Giovannantonio et al., 2021). This increases the expression of OCT4, which together with NANOG and its downstream target ESRRB, provide positive regulatory inputs into the PGC GRN (centered on the TFs Blimp1, Prdm14, and Ap2γ), sufficient to drive PGCLC differentiation in the absence of cytokines. (C) If ESRRB is overexpressed, there is no repression of *Otx2*, and the OTX2-mediated repression of *Nanog* and *Oct4* remains in place, blocking PGCLC differentiation. (D) In *Otx2*-heterozygous EpiLCs, repression of *Nanog* and *Oct4* by OTX2 is diminished in strength. This enables induction of ESRRB to provide sufficient input into the PGC GRN (alongside mild inputs from NANOG and OCT4) to direct PGCLC differentiation.

In wild-type EpiLCs, PGC-promoting cytokines suppress *Otx2* expression and activate the PGC-specific gene regulatory network (GRN) (Zhang et al., 2018a). Our present findings, conducted in the absence of PGC-promoting cytokines, can be incorporated into current thinking about EpiLC-PGCLC differentiation as shown (Figure 4). In the absence of cytokines, OTX2 protein levels are sufficient to block activation of the PGCLC program. Recent findings indicate that this effect of OTX2 on the PCG program results largely from direct repression of *Nanog* and *Oct4* (Figure 4A) (Di Giovannantonio et al., 2021). Induction of transgenic Nanog in EpiLCs shifts the balance between the mutual antagonists NANOG and OTX2 in favor of NANOG (Figure 4B). Once OTX2 levels are sufficiently decreased, this enables NANOG to act on regulatory elements controlling genes in the PGC-specific GRN, including *Blimp1*, *Prdm14*, and *Ap2γ*, as previously suggested (Murakami et al., 2016). In addition, NANOG can activate *Esrrb*, which may also positively affect the PGC-specific GRN. Moreover, a reduction in OTX2 eliminates suppression of *Oct4*, which can also positively feed into the PGC-specific GRN (Figure 4B). If instead transgenic Esrrb is induced, a positive effect on the PGC-specific GRN can also be envisaged (Figure 4C). However, in this case, *Otx2* is not suppressed, OTX2 remains high, suppression of *Nanog* and *Oct4* is maintained, and the positive input into the PGC-specific GRN is insufficient to activate PGCLC differentiation. In contrast, in *Otx2*^+/−^ cells, the balance between the mutual antagonists OTX2 and NANOG shifts (Figure 4D). In these conditions, induction of ESRRB may provide a sufficient positive effect on the PGC-specific GRN to direct some PGC differentiation due to a weakened suppression of *Nanog* and *Oct4* by OTX2. Further work will be required to bring greater clarity to the early events involved in germline specification prior to activation of the PGC-specific GRN.

## Experimental procedures

### PGCLC differentiation

Cytokine-free PGCLC differentiation with or without 1 μg/mL doxycycline was performed as previously described (Zhang et al., 2018a).

Additional methods can be found in the Supplemental experimental procedures.

### Data and code availability

Code and data to reproduce the analysis and figures are available at https://github.com/MatusV8/Otx2het.

## Author contributions

Conceptualization, M.V., J.Z., M.Z., and I.C.; Methodology, M.V., J.Z., and M.Z.; Investigation, M.V., J.Z., J.S., and M.Z.; Formal Analysis, M.V. and M.Z.; Writing – Original Draft, M.V., M.Z., and I.C.; Writing – Review & Editing, M.V., M.Z., and I.C.; Visualization, M.V.; Supervision, I.C. and M.Z.; Resources, I.C.; Funding Acquisition, M.Z. and I.C.

## Conflict of interests

The authors declare no competing interests.

## Supplemental information

Document S1. Figures S1–S3, Table S1, and Supplemental experimental procedures

Document S2. Article plus supplemental information
