# Supplementary material for: Differential repression of Otx2 underlies the capacity of NANOG and ESRRB to induce germline entry
Source: Stem Cell Reports. 2021 Dec 30;17(1):35–42. doi: 10.1016/j.stemcr.2021.11.013 (PMC8758940; doi:10.1016/j.stemcr.2021.11.013)
Supplement: Document S1. Figures S1–S3, Table S1, and Supplemental experimental procedures [file mmc1.pdf]

Stem Cell Reports, Volume *17*

Supplemental Information

Differential repression of *Otx2* underlies the capacity of NANOG and ESRRB to induce germline entry

Matúš Vojtek, Jingchao Zhang, Juanjuan Sun, Man Zhang, and Ian Chambers

Figure S1 (related to figure 1)

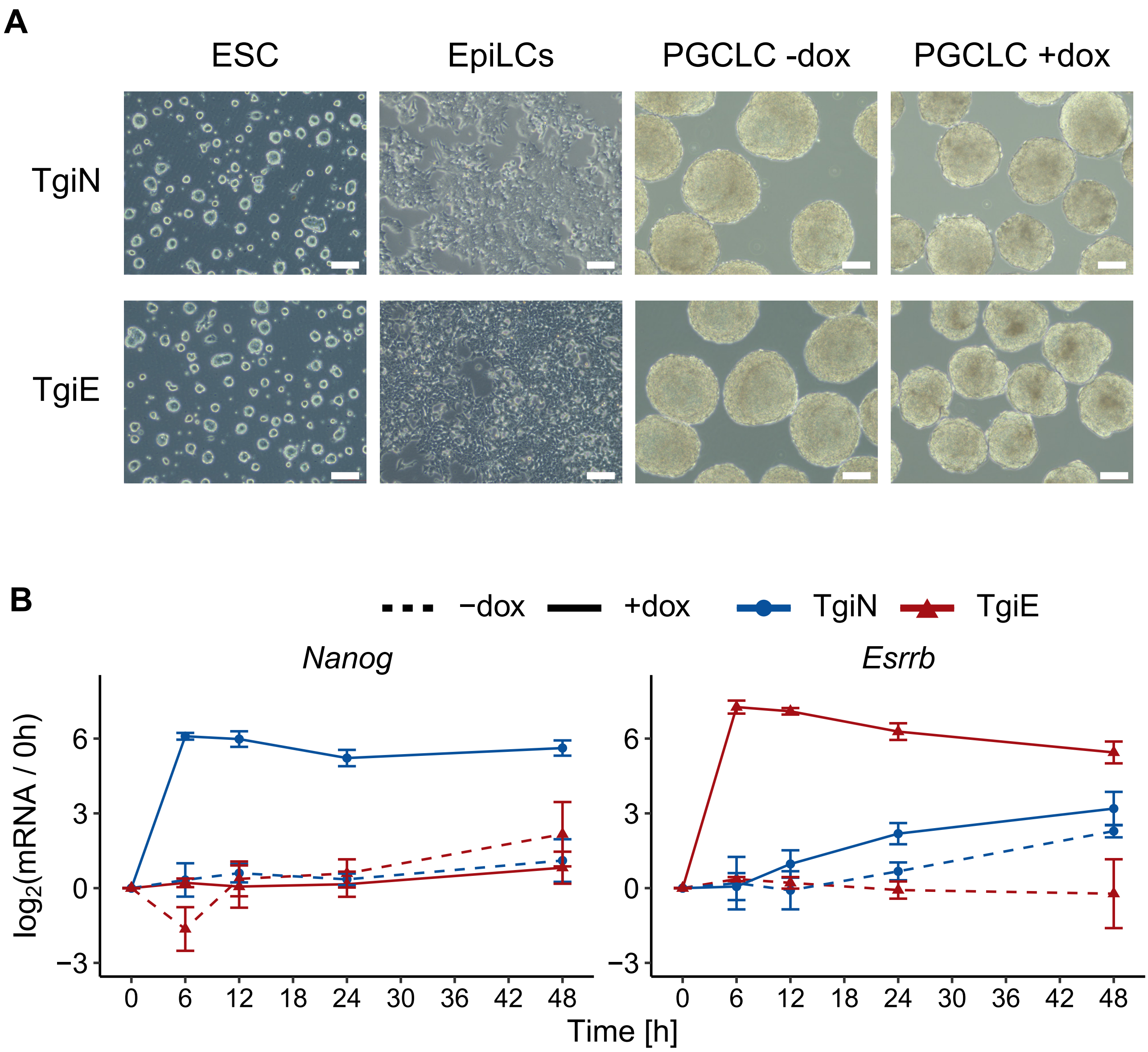

Figure S1: Validation of doxycycline inducible Nanog and Esrrb cell lines.
A) Photographs of TgiN and TgiE ESCs, EpiLCs and cell aggregates at day 6 of cytokine-free PGCLC differentiation in the presence (+dox) or absence (-dox) of doxycycline. White bars represent 100 µm.
B) Relative changes of *Esrrb* and *Nanog* mRNAs after aggregation of E14Tg2a TetON-Nanog (TgiN) and E14Tg2a TetON-Esrrb (TgiE) EpiLCs cultured in the absence (-dox) or presence (+dox) of doxycycline at the indicated time point. Lines, points and triangles represent mean log_2 fold-change (FC) differences between data points and the zero-time timepoint (mean ± SD, n = 3 independent experiments).

Figure S2 (related to figure 2)

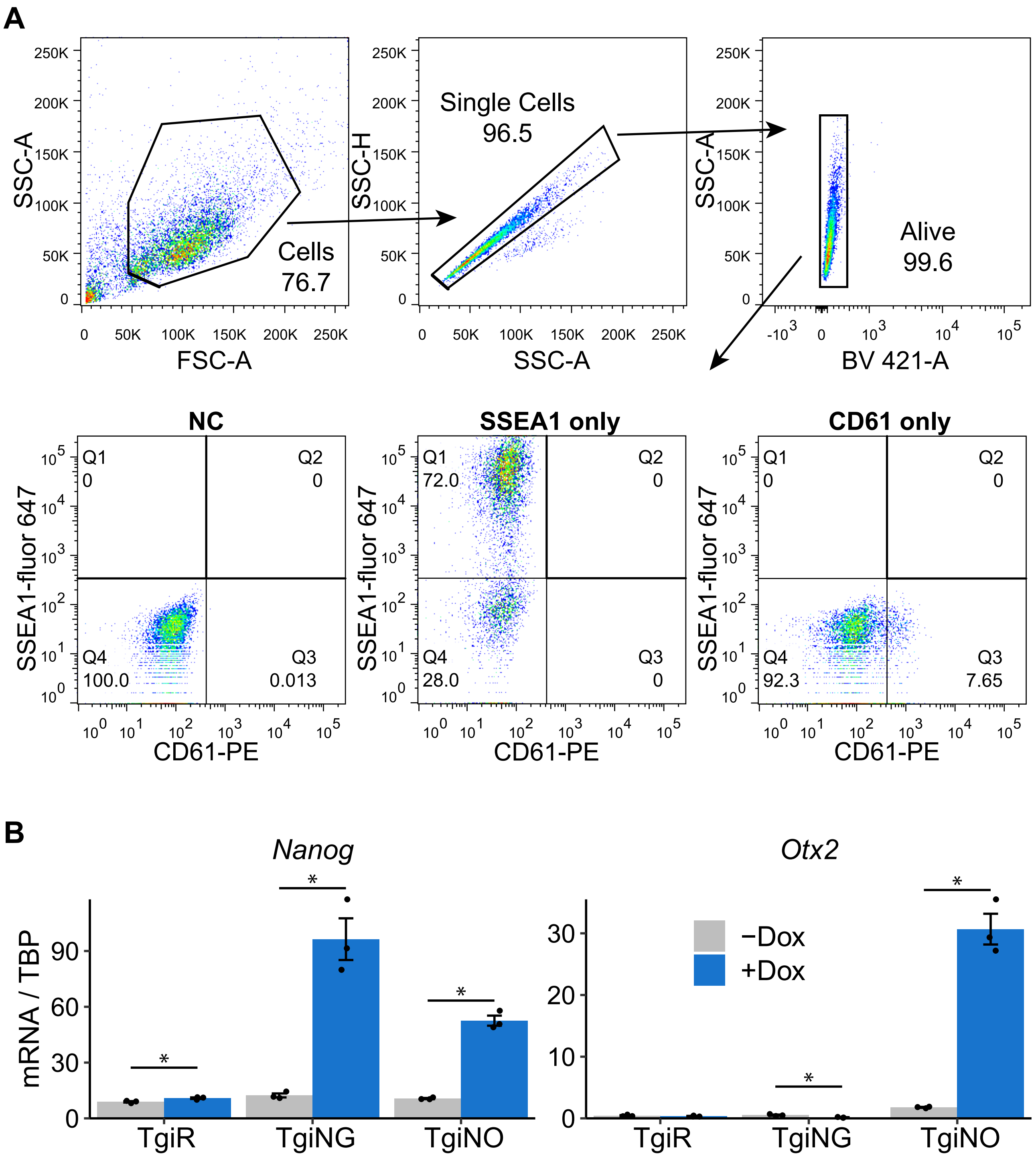

Figure S2: Gating strategy and activity of transgenes in TgiNG and TgiNO ESCs.
A) Gating strategy to quantify SSEA1+CD61+ cells. Single and living cells were assessed for fluor 647 and PE signal. Unstained negative control (NC), and single stained controls were used to draw gates. **B)** RT-qPCR quantification of *Nanog* and *Otx2* mRNAs in TgiR, TgiNG and TgiNO ESCs (related to Figure 1A) before and after 48 hours of doxycycline treatment. Bars are mean ± SEM, points are individual data measurements (n = 3 independent experiments). * $p < 0.05$ (t-test).

Figure S3 (related to figure 3)

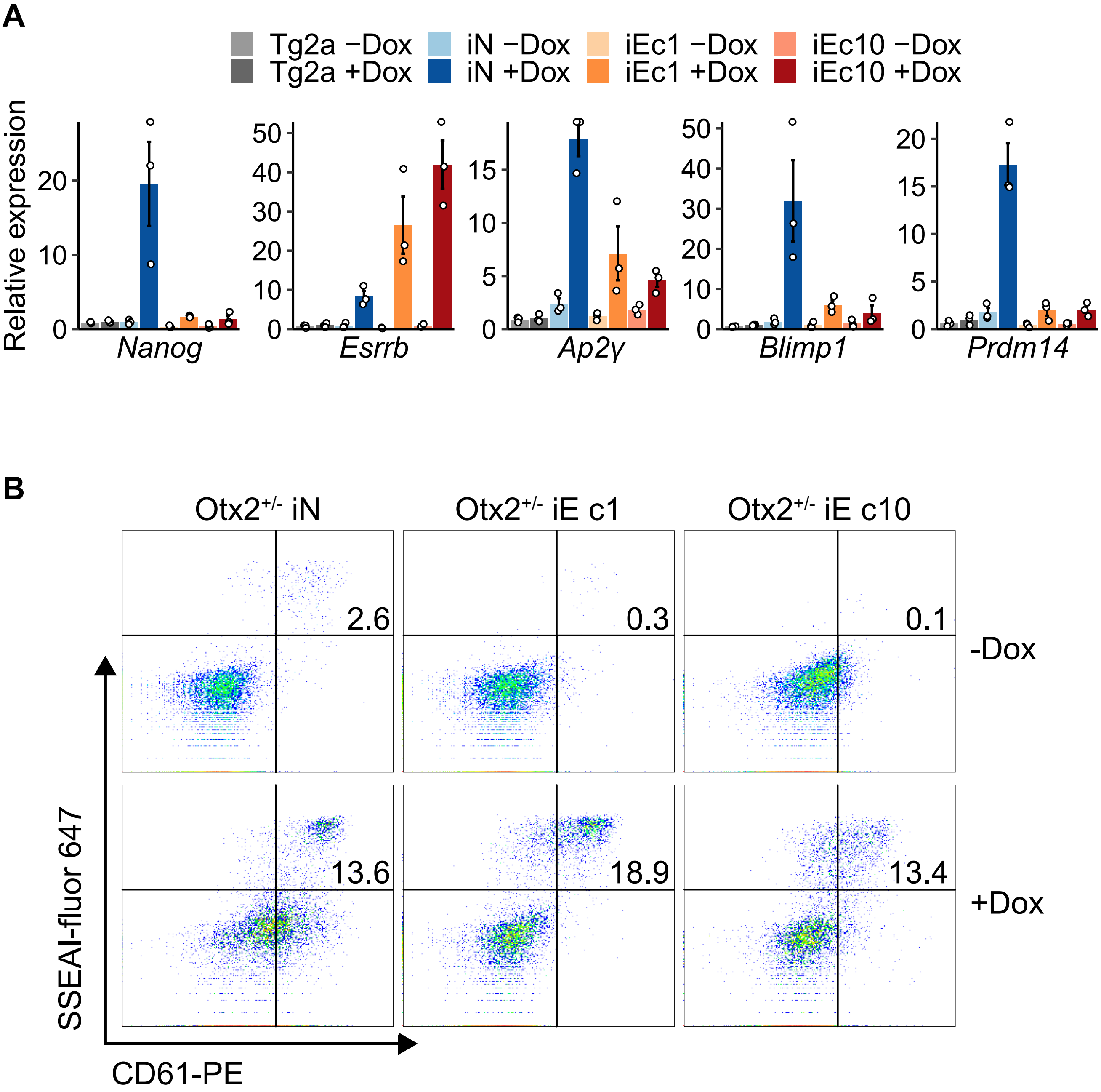

Figure S3: Cytokine-free differentiation of Nanog and Esrrb inducible *Otx2*$^{+/-}$ cell lines.
A) qRT-PCR analysis of the indicated mRNAs at day 2 of the cytokine-free PGCLC differentiation in the presence (+Dox) or absence (-Dox) of doxycycline. Bars are mean ± SEM, points are individual data measurements (n = 3 independent experiments) of relative expression normalised to the mean expression in Tg2a -Dox sample for each mRNA. **B)** Representative flow cytometry analysis of SSEA1 and CD61 expression at day 6 of cytokine-free PGCLC differentiation of the indicated cell lines in the presence (+Dox) or absence (-Dox) of doxycycline. Percentages of SSEA1+CD61+ populations are indicated.

Table S1: Primers used for qRT-PCR

Name	Forward primer (5' -> 3')	Reverse primer (5' -> 3')
Tbp	GGGGAGCTGTGATGTGAAGT	CCAGGAAATAATTCTGGCTCA
Pou5f1	CCCCAATGCCGTGAAGTTG	TCAGCAGCTTGGCAAACTGTT
Nanog	AGGATGAAGTGCAAGCGGTG	TGCTGAGCCCTTCTGAATCAG
Esrrb	CGATTCATGAAATGCCTCAA	CCTCCTCGAACTCGGTCA
Fgf5	AAAACCTGGTGCACCCTAGA	CATCACATTCCCGAATTAAGC
Otx2	GACCCGGTACCCAGACATC	GCTCTTCGATTCTTAAACCATACC
Pou3f1	CCATCGAGGTGGGTGTCAAA	CCTCCTTCTCCAGTTGCAGG
Blimp1	CCCCTCATCGGTGAAGTCTA	TGGTGGAACTCCTCTCTGGA
Prdm14	TCAATTCACTCCCGAAGTACCA	CCGGGGATGGCAGAAGTAAA
Ap2γ	ATCCCTCACCTCTCCTCTCC	CCAGATGCGAGTAATGGTCGG

Supplemental experimental procedures

Cell culture

All ESCs were derived from E14Tg2a (Tg2a) mouse ESCs (1) and were routinely cultured in Glasgow Minimum Essential Medium (GMEM; Sigma, cat. n. G5154) supplemented with 10% FCS (Gibco cat. n. 10270-106), 100 U/ml LIF (homemade), Non-essential amino acids (Invitrogen, cat. n. 11140-036), 1 mM sodium pyruvate (Invitrogen, cat. n. 11360-039), 2 mM L-glutamine (Invitrogen, cat. n. 25030-024) and 50 nM 2-mercaptoethanol (Gibco, cat. n. 31350-010) at density of 3-10x10^5 cells per cm^2 in culture flasks coated with 0.1% gelatine as previously described (2). Presence of mycoplasma contamination was routinely tested by MycoAlert™ Mycoplasma Detection Kit (Lonza; cat. n. LT07-318).

Generation of doxycycline inducible cell lines

To generate *Otx2*$^{+/+}$ Nanog or Esrrb inducible cell lines (TgiN, TgiE), firstly CAG-rtTAms2 was integrated into the *Rosa26* locus of E14Tg2a ESCs as described previously (3). E14Tg2a-rtTAms2 cells were transfected with piggyBac-TetON-Nanog-PGK-Hph-pA or piggyBac-TetON-Esrrb-PGK-Hph-pA together with pCMV-hyPBASE (4) plasmids using lipofectamine 3000 (Thermofisher; cat. n. L3000015).

To generate TgiNG and TgiNO cells, E14Tg2a-rtTAms2 cells were transfected with piggyBac-TetO-FRT5-TdTomato-2a-HygR-FRT3-tk (5) and selected for clones with robust TdTomato expression in presence of doxycycline and low levels of TdTomato in the absence of doxycycline. To integrate Nanog-T2A-GFP and Nanog-T2A-Otx2 cassettes, *Rosa26*: rtTAms2; E14Tg2a-TetON-TdTomato ESCs were transfected with pShuttle-TetON-FRT5-$(Flag)_3$-Nanog-T2A-GFP-IRES-PURO-FRT3 or pShuttle-TetON-FRT5-$(Flag)_3$-Nanog-T2A-Otx2-IRES-PURO-FRT3 together with pPGK-FLPobpa (Adgene #13793) using Lipofectamine 3000 (Thermofisher; cat. n. L3000015). Cells resistant to puromycin and without TdTomato expression in presence of doxycycline were selected.

To generate *Otx2*$^{+/-}$ ΔPE::GFP ESCs with doxycycline inducible transgenes, *Otx2*$^{lacZ/fl}$ ΔPE::GFP c11 ESCs (6) were transfected with piggyBac-TetON-Nanog-PGK-Hph-pA or piggyBac-TetON-Esrrb-PGK-Hph-pA together with pCMV-hyPBASE (4) and pCAG-rTtAsm2-IRES-BSD using lipofectamine 3000 (Thermofisher; cat. n. L3000015). Cells were selected with hygromycin and blasticidin for twelve days and single clones were picked. Two clones for Esrrb and one for Nanog were selected with capacity to induce the transgene and which express similar levels of *Otx2* to the parental cell line.

Flow cytometry

Cell aggregates were collected, washed with PBS and dissociated in 0.05 % Trypsin for ~10 minutes at 37 °C. Trypsin was neutralised with MEF medium [Glasgow Minimum Essential Medium (GMEM; Sigma, cat. n. G5154) supplemented with 10% FCS (Gibco cat. n. 10270-106), Non-essential amino acids (Invitrogen, cat. n. 11140-036), 1 mM sodium pyruvate (Invitrogen, cat. n. 11360-039), 2 mM L-glutamine (Invitrogen, cat. n. 25030-024) and 50 nM 2-mercaptoethanol (Gibco, cat. n. 31350-010)] and cells were passed through a cell strainer. Samples (~15%) from each cell suspension were combined in a separate tube for control samples. Cells were collected by centrifugation (3 min at 300g) and resuspended in 100 µl of MEF medium supplemented with 1:200 v/v Alexa Fluor 647 anti-mouse/human CD15 (SSEA-1) (Biolegend, cat. n. 125608) and 1:500 v/v PE anti-mouse/rat CD61 (Biolegend, cat. n. 104307). The control cells were resuspended in 300 µl of MEF medium and cell suspension was divided into three 100 µl parts. SSEA-I and CD61 antibodies were added to one fraction resulting in two single-stained control samples and one unstained control. Cells were incubated for 15 min at room temperature and washed twice with PBS before analysis at BD Fortessa 5 laser system. Flow cytometry data were analysed using FlowJo X 0.7 software. Live cells were gated based on DAPI signal, SSEA-I+CD61+ populations were gated based on the negative and single stain controls (see Figure S2).

qRT-PCR

Single cell suspension was collected by centrifugation (3 min at 300 g) and total RNA was extracted by Illustra™ RNAspin RNA Isolation Kit (GE Healthcare, cat. n. GE25-0500-72) according to manufacturer instructions. First strand cDNA was synthetised using 200-1000 ng RNA, SuperScript III reverse transcriptase (Thermofisher, cat. n. 18080044) and random hexamers at the final concentration 2.5 mM. cDNA was diluted 1:10 in nuclease-free grade water. qPCR mix was prepared by mixing 5 µl cDNA, 4.5 µl of 2x Takyon SYBR Green master mix (Eurogentec, cat. n. UF-NSMT-B0701) and 0.5 µl of 10 mM mixture of a primer pair (Table S1). Specificity and efficiency of used primer pairs were tested prior the experiments. Quantitative polymerase reactions were performed in two technical replicates for each independent replicate using LightCycler 450 instrument (Roche) with program: 3 min at 95 ºC, 45x (10s at 95 ºC, 20s at 60 ºC, 30s at 72 ºC). The cycle corresponding to the crossing point (Cp) was determined using the second derivative method and values for technical replicates were

averaged. Relative mRNA levels for each sample were determined by $2^{-\Delta\Delta Cp}$ method (7) using *Tbp* as a reference gene. For figures 1F, 2B and S1B, relative mRNA expression was normalised by calculating log2 of fold change between each time point and the 0h time point for each replicate. For figures 3B and S3A, values were normalised by diving each value by the mean value of the reference samples (*Otx2*$^{+/+}$ and Tg2a -Dox respectively).

Statistical analysis and data visualisation
Statistical analysis and visualisation was done using R v4.0.3 (8) and tidyverse packages (9). The number of independent experiments performed on different days (n) underlying each plot is reported in the figure legends. Summary statistics was reported as mean value ± standard error of the mean (SEM) or standard deviation (SD). Two-tailed t-test was used to compare treatment samples to the reference sample. Benjamin-Hochberg method was used to adjust p-values to correct for multiple testing.

References

1. Hooper M, Hardy K, Handyside A, Hunter S, Monk M. HPRT-deficient (Lesch–Nyhan) mouse embryos derived from germline colonization by cultured cells. Nature. 1987 Mar;326(6110):292–5.

2. Smith AG. Culture and differentiation of embryonic stem cells. Journal of Tissue Culture Methods. 1991 Jun 1;13(2):89–94.

3. Bressan RB, Dewari PS, Kalantzaki M, Gangoso E, Matjusaitis M, Garcia-Diaz C, et al. Efficient CRISPR/Cas9-assisted gene targeting enables rapid and precise genetic manipulation of mammalian neural stem cells. Development. 2017 Feb 15;144(4):635–48.

4. Yusa K, Zhou L, Li MA, Bradley A, Craig NL. A hyperactive piggyBac transposase for mammalian applications. Proc Natl Acad Sci U S A. 2011 Jan 25;108(4):1531–6.

5. Festuccia N, Osorno R, Halbritter F, Karwacki-Neisius V, Navarro P, Colby D, et al. Esrrb Is a Direct Nanog Target Gene that Can Substitute for Nanog Function in Pluripotent Cells. Cell Stem Cell. 2012 Oct;11(4):477–90.

6. Zhang J, Zhang M, Acampora D, Vojtek M, Yuan D, Simeone A, et al. OTX2 restricts entry to the mouse germline. Nature. 2018 Oct;562(7728):595–9.

7. Livak KJ, Schmittgen TD. Analysis of Relative Gene Expression Data Using Real-Time Quantitative PCR and the 2−ΔΔCT Method. Methods. 2001 Dec 1;25(4):402–8.

8. Team RC, others. R: A language and environment for statistical computing. 2013;

9. Wickham H, Averick M, Bryan J, Chang W, McGowan LD, François R, et al. Welcome to the Tidyverse. Journal of Open Source Software. 2019 Nov 21;4(43):1686.
