# Supplementary material for: Differential repression of Otx2 underlies the capacity of NANOG and ESRRB to induce germline entry
Source: Stem Cell Reports. 2021 Dec 30;17(1):35–42. doi: 10.1016/j.stemcr.2021.11.013 (PMC8758940; doi:10.1016/j.stemcr.2021.11.013)
Supplement: Document S2. Article plus supplemental information [file mmc2.pdf]

Stem Cell Reports
Report
OPEN ACCESS

Differential repression of *Otx2* underlies the capacity of NANOG and ESRRB to induce germline entry

Matúš Vojtek,[1] Jingchao Zhang,[1,5] Juanjuan Sun,[2,4] Man Zhang,[1,2,3,4,*] and Ian Chambers[1,*]
[1]Centre for Regenerative Medicine, Institute for Stem Cell Research, School of Biological Sciences, University of Edinburgh, 5 Little France Drive, Edinburgh EH16 4UU, Scotland
[2]Center for Cell Lineage and Atlas (CCLA), Bioland Laboratory, Guangzhou Regenerative Medicine and Health Guangdong Laboratory, Guangzhou, China
[3]The Fifth Affiliated Hospital of Guangzhou Medical University, Guangzhou, China
[4]Guangzhou Laboratory, No. 9 XingDaoHuanBei Road, Guangzhou International Bio Island, Guangzhou, 510005, Guangdong Province, China
[5]Present address: University of Pennsylvania, Perelman School of Medicine, Smilow Center for Translational Research, 3400 Civic Center Boulevard, Philadelphia, PA 19104-5157, USA
*Correspondence: zhang_man@gzlab.ac.cn (M.Z.), ichambers@ed.ac.uk (I.C.)
https://doi.org/10.1016/j.stemcr.2021.11.013

SUMMARY

Primordial germ cells (PGCs) arise from cells of the post-implantation epiblast in response to cytokine signaling. PGC development can be recapitulated *in vitro* by differentiating epiblast-like cells (EpiLCs) into PGC-like cells (PGCLCs) through cytokine exposure. Interestingly, the cytokine requirement for PGCLC induction can be bypassed by enforced expression of the transcription factor (TF) NANOG. However, the underlying mechanisms are not fully elucidated. Here, we show that NANOG mediates *Otx2* downregulation in the absence of cytokines and that this is essential for PGCLC induction by NANOG. Moreover, the direct NANOG target gene *Esrrb*, which can substitute for several NANOG functions, does not downregulate *Otx2* when overexpressed in EpiLCs and cannot promote PGCLC specification. However, expression of ESRRB in *Otx2*$^{+/-}$ EpiLCs rescues emergence of PGCLCs. This study illuminates the interplay of TFs occurring at the earliest stages of PGC specification.

INTRODUCTION

Germline development and sexual reproduction depend on the establishment of primordial germ cells (PGCs). In mice, PGCs are induced mainly by bone morphogenic factor 4 (BMP4) and other cytokines, including BMP8a and BMP2, that act on proximal posterior epiblast cells at embryonic day 6 (Hayashi et al., 2007; Lawson et al., 1999). Prospective PGCs downregulate the epiblast transcription factor (TF) OTX2 and subsequently activate the key PGC TFs BLIMP1 (PRDM1), PRDM14 and AP2γ (Kurimoto et al., 2008; Ohinata et al., 2005; Vincent et al., 2005; Weber et al., 2010; Yamaji et al., 2008; Zhang and Chambers, 2019). These events can be recapitulated *in vitro* by differentiating naive embryonic stem cells (ESCs) into epiblast-like cells (EpiLCs), which are transiently competent to specify PGC-like cells (PGCLCs) in response to BMP4 and associated cytokines (Hayashi et al., 2011; Hayashi and Saitou, 2013). The requirement for BMP4 and associated cytokines can however be bypassed by induction of NANOG in EpiLCs (Murakami et al., 2016). Combined ectopic expression of NANOG and cytokine treatment further expands the proportion of PGCLCs in the differentiated population (Murakami et al., 2016). Even though *Nanog* is not essential for the emergence of PGCs, or for germline transmission (Carter et al., 2014; Zhang et al., 2018b), *Nanog* deletion results in a large decrease in PGC numbers both *in vitro* and *in vivo* (Chambers et al., 2007; Murakami et al., 2016; Yamaguchi et al., 2009; Zhang et al., 2018b).

The target genes through which NANOG acts in ESCs have been identified and include *Esrrb* and *Otx2*, which are regulated positively and negatively by NANOG, respectively (Festuccia et al., 2012). Both of these genes also regulate the PGC compartment (Mitsunaga et al., 2004; Zhang et al., 2018a). Germline loss of function for *Esrrb* results in a similar quantitative reduction in PGC numbers at midgestation, as seen when *Nanog* is deleted specifically from the germline (Mitsunaga et al., 2004; Zhang et al., 2018b). Moreover, deletion of *Nanog* impairs induction of PGCLC differentiation in response to PGC-promoting cytokines (Murakami et al., 2016). This absence of PGC differentiation can be compensated for by enforced expression of ESRRB (Zhang et al., 2018b). Consistent with a conserved epistatic relationship between Nanog and Esrrb both in the preimplantation epiblast and in the germline, knockin of *Esrrb* to the *Nanog* locus overcomes the reduction in PGC numbers resulting from germline-specific *Nanog* deletion (Zhang et al., 2018b).

In ESCs, Otx2 and Nanog antagonize each other by mutual repression (Acampora et al., 2017; Festuccia et al., 2012). We have shown that entry to the germline is blocked when OTX2 expression is maintained during the first 2 days of EpiLC-PGCLC differentiation (Zhang et al., 2018a; Zhang and Chambers, 2019). In contrast, *Otx2* deletion dramatically increases PGCLC numbers *in vitro* and raises PGC numbers *in vivo* (Zhang et al., 2018a). Recently, OTX2 has been shown to act through *cis*-acting binding sites that repress transcription of *Nanog* and *Pou5f1* (*Oct4*) (Di

© 2021 The Author(s).
This is an open access article under the CC BY license (http://creativecommons.org/licenses/by/4.0/).

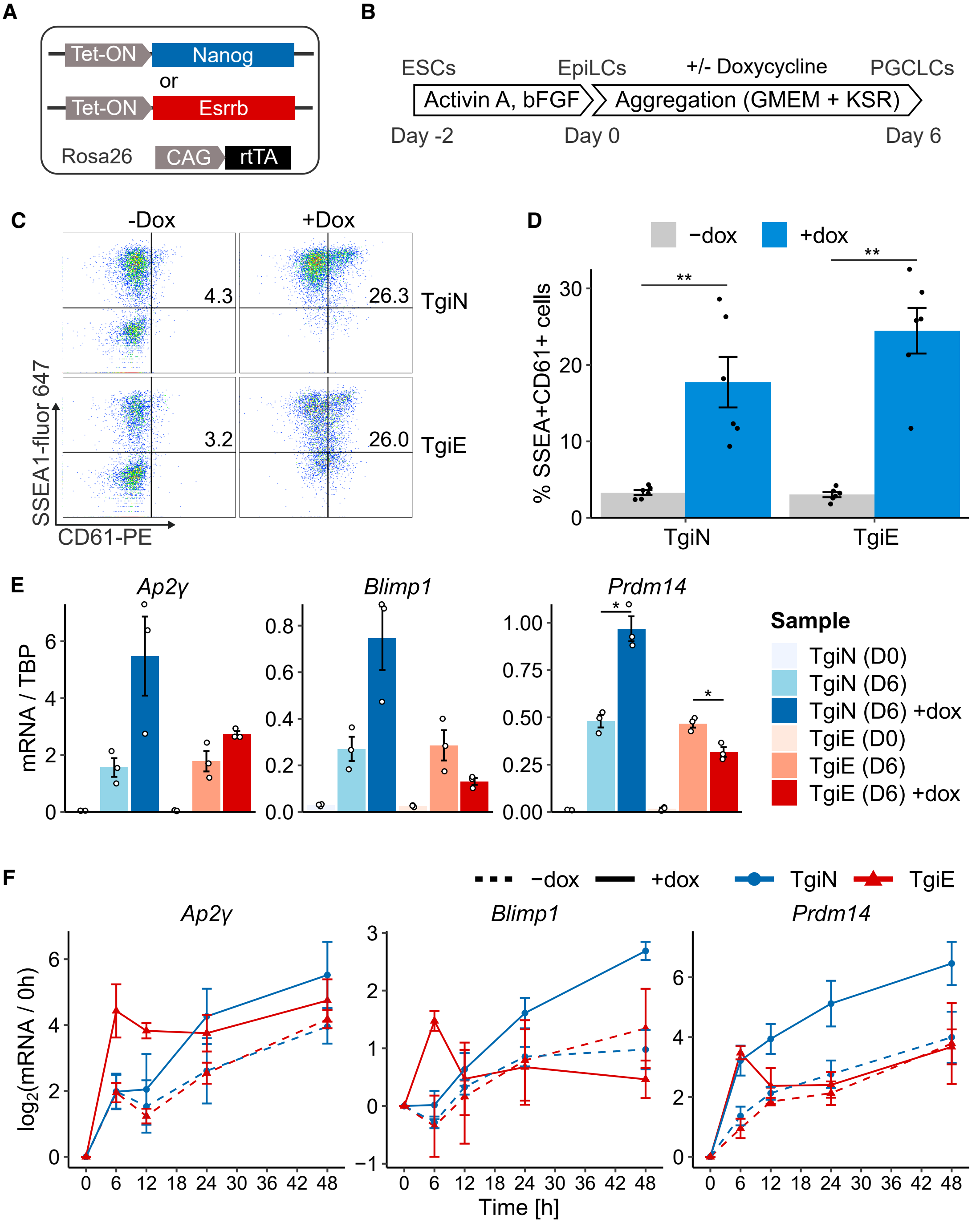

Figure 1. ESRRB cannot activate the PGC program

(A) Cartoon of TetON-Nanog (TgiN) and TetON-Esrrb (TgiE) cell lines. TetON expression cassettes were randomly integrated into E14Tg2a cells expressing rtTA from *Rosa26*.

(B) The cytokine-free PGCLC differentiation protocol. ESCs cultured in 2i/LIF were differentiated to EpiLCs by culture in Activin A and bFGF for 2 days. EpiLCs were then aggregated in GMEM+KSR medium for 6 days with (+dox) or without (−dox) doxycycline, and PGCLC status was analyzed.

(C) Representative example of flow cytometry analysis of PGCLC aggregates from TgiN and TgiE cells at day 6 with or without dox, showing the percentage of $SSEA1^{+}CD61^{+}$ cells.

(D) Quantification of (C). Bars are mean ± SEM, points are individual data measurements (n = 6 independent experiments).

(legend continued on next page)

Giovannantonio et al., 2021). However, despite increasing knowledge of the relationships among ESRRB, NANOG, and OTX2, the interplay among these factors in PGC induction is not fully understood. Here we assess the capacity of ESRRB and NANOG to induce PGCLC differentiation using inducible transgene systems in the absence of cytokines. Our results uncover a differential capacity of NANOG and ESRRB to repress *Otx2* and an OTX2 dose-dependent barrier to germline induction by ESRRB and NANOG in the absence of cytokines.

RESULTS

ESRRB cannot activate the PGCLC program

To examine whether ESRRB can induce cytokine-free PGCLC differentiation similarly to NANOG, we overexpressed NANOG or ESRRB during cytokine-free PGCLC differentiation. Cell lines carrying tetracycline-inducible Nanog (TgiN) or Esrrb (TgiE) transgenes were generated by integrating piggyBac transposons into E14Tg2a ESCs expressing the modified reverse tetracycline transactivator (rtTA2) (Urlinger et al., 2000) from *Rosa26* (Figure 1A). The resulting TgiN and TgiE ESCs were then differentiated to PGC-competent epiblast-like cells (Figure 1B). In the original PGCLC differentiation protocol, EpiLCs are aggregated in a cytokine cocktail that is required for PGCLC specification (Hayashi et al., 2011; Hayashi and Saitou, 2013). However, NANOG can direct PGCLC differentiation in the absence of cytokines (Murakami et al., 2016). We therefore omitted cytokines and tested the ability of NANOG or ESRRB overexpression to induce PGCLC development (Figure 1B). TgiN and TgiE cells formed similar colonies both as naive ESCs and EpiLCs (Figure S1A). Doxycycline addition during PGCLC specification from EpiLCs activated robust induction of Nanog or Esrrb transgenes in TgiN or TgiE cells, respectively (Figure S1B). Induced expression of either *Nanog* or *Esrrb* resulted in similar levels of surface expression of SSEA1 and CD61, which jointly mark PGCLCs (Hayashi et al., 2011) (Figures 1C, 1D, and S2A). However, in contrast to NANOG, induction of ESRRB failed to increase expression of *Blimp1* or *Prdm14* mRNAs and showed only a modest increase in *Ap2*γ mRNA (Figure 1E). We therefore assessed changes in PGC TF expression earlier, during the first 48 h of differentiation (Figures 1F and S1B). Induction of NANOG activated expression of both *Esrrb* and the PGC transcription factors *Blimp1*, *Prdm14*, and *Ap2*γ. Interestingly, while ESRRB induction increased *Blimp1*, *Prdm14*, and *Ap2*γ mRNAs within the first 6 h of differentiation, ESRRB did not sustain *Blimp1* and *Prdm14* expression at later times (Figure 1F). Therefore, ESRRB unlike NANOG does not produce a sustained activation of the PGC program during cytokine-free PGCLC differentiation.

NANOG induces PGCLCs by repressing *Otx2*

Our previous results show that the requirement of cytokines for PGCLC formation is also eliminated in *Otx2*$^{-/-}$ cells (Zhang et al., 2018a). OTX2 and NANOG have antagonistic functions in ESCs (Acampora et al., 2017). Moreover, NANOG can directly downregulate *Otx2* in ESCs (Figure 2A; Festuccia et al., 2012; Heurtier et al., 2019). Microarray data suggest that this capacity to repress *Otx2* may not be shared by ESRRB (Figure 2A; Festuccia et al., 2012). This raises the hypothesis that ESRRB cannot effectively induce PGCLC specification because of an impaired capacity to repress *Otx2*. To address this, we quantified *Otx2* mRNA during the first 48 h of EpiLC aggregation of TgiN and TgiE cells in cytokine-free medium (Figure 1B). As expected, NANOG induction drove a rapid decrease in *Otx2* between 6 and 12 h compared with uninduced cells (Figure 2B). In contrast, ESRRB induction did not affect *Otx2* mRNA levels during the first 24 h (Figure 2B).

We next tested whether OTX2 clearance is necessary for NANOG to induce PGCLCs. To do this, we generated E14Tg2a ESC lines that can induce either GFP (TgiNG) or OTX2 (TgiNO) from the same transgene that induces NANOG (Figure 2C). TgiNG and TgiNO cells were generated by replacing TdTomato in E14Tg2a TetON-TdTomato (TgiR) cells with cassettes encoding Nanog-t2a-GFP or Nanog-t2a-Otx2 (Figure 2C). Doxycycline treatment increased *Nanog* expression ~8- and 5-fold in TgiNG and TgiNO cells, respectively (Figure S2B). In addition, *Otx2* mRNA was induced by doxycycline only in TgiNO cells (Figure S2B). To assess the effect of OTX2 on the ability of NANOG to induce PGCLC differentiation, TgiNG and TgiNO cells were subjected to PGCLC differentiation in the absence of cytokines. Simultaneous induction of GFP and NANOG upregulated expression of PGCLC surface markers CD61 and SSEA1 (Figures 2D and 2E). In contrast, simultaneous induction of OTX2 and NANOG markedly

(E) qRT-PCR of indicated transcripts in TgiN and TgiE cells at day 6 of cytokine-free PGCLC differentiation with or without dox. Bars represent mean mRNA levels normalized to *Tbp* mRNA. Bars are mean ± SEM, and points are individual data measurements (n = 3 independent experiments).
(F) Time-course analysis of the indicated mRNAs during the first 48 h of cytokine-independent PGCLC differentiation of TgiN and TgiE EpiLCs with or without dox. Points, triangles, and lines represent the mean $\log_2$ fold change in ratio between *Tbp*-normalized expression and the zero time point (mean ± SD; n = 3 independent experiments).
*p < 0.05 and **p < 0.01 (t test).

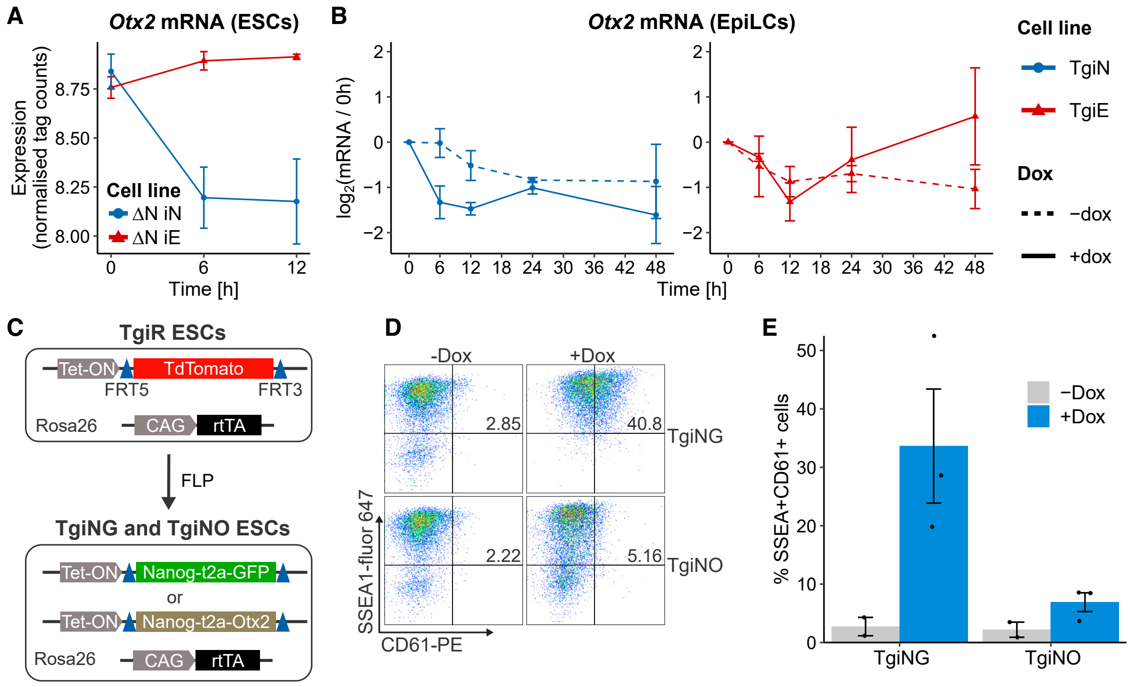

Figure 2. *Otx2* downregulation is essential for PGCLC induction by NANOG

(A) Relative changes of *Otx2* mRNA in *Nanog*$^{-/-}$ TetON-Nanog (ΔN-iN) or *Nanog*$^{-/-}$ TetON-Esrrb (ΔN-iE) ESCs at indicated time points after doxycycline treatment (mean ± SD, n = 3 independent experiments). Data adapted from Festuccia et al. (2012).

(B) Relative changes of *Otx2* mRNA after aggregation of TgiN and TgiE EpiLCs cultured with (+dox) or without (−dox) doxycycline. Lines, points, and triangles represent the mean $\log_2$ in ratio between *Tbp*-normalized expression and the zero time point (mean ± SD; n = 3 independent experiments).

(C) *Rosa26*: rtTA; E14Tg2a-TetON-TdTomato (TgiR) ESCs were modified as shown to derive inducible Nanog-t2a-GFP (TgiNG) or Nanog-t2a-Otx2 (TgiNO) ESCs.

(D) Representative example of flow cytometry analysis of PGCLC aggregates from TgiNG and TgiNO cells at day 6 with or without dox. Percentages of SSEA1^{+}CD61^{+} cells are shown.

(E) Quantification of (D). Bars are mean ± SEM, points are individual data measurements (n = 2 independent experiments for −dox, and n = 3 for +dox).

reduced the population of SSEA1^{+}/CD61^{+} cells (Figures 2D and 2E). This indicates that the capacity of NANOG to function in PGCLC induction requires repression of *Otx2*.

Otx2 heterozygosity enables ESRRB to induce cytokine-free PGC differentiation

To test whether OTX2 is a limiting factor that prevents ESRRB from activating PGCLC specification, we integrated doxycycline-inducible Nanog (iN) or Esrrb (iE) transgenes into heterozygous *Otx2*$^{lacZ/fl}$ ESCs (Acampora et al., 2013; Zhang et al., 2018a) (Figure 3A). This cell line also contains a GFP transgene that reports the activity of the *Oct4* distal enhancer (ΔPE::GFP) (Figure 3A) which becomes activated in PGCLCs (Magnusdottir et al., 2013). We refer to these cells as *Otx2*$^{+/-}$ iE and *Otx2*$^{+/-}$ iN cells. We isolated two *Otx2*$^{+/-}$ iE clones (1 and 10) and one *Otx2*$^{+/-}$ iN clone that each express approximately 50% the level of *Otx2* mRNA compared with *Otx2*$^{+/+}$ cells in the EpiLC state (Figure 3B).

Doxycycline treatment of *Otx2*$^{+/-}$ iN and *Otx2*$^{+/-}$ iE cell lines increased expression of the transgenes by 20- to 40-fold compared with wild-type cells at day 2 of cytokine-free PGCLC differentiation (Figure S3A). When *Otx2*$^{+/-}$ cell lines were subjected to cytokine-free PGCLC induction in the absence of doxycycline, surface expression of CD61/SSEA1 was not induced in iE or iN cell lines (Figure S3B). However, upon induction of either NANOG or ESRRB by doxycycline, surface expression of CD61/SSEA1 was induced (Figures 3C and S3B). In addition, the proportion of cells expressing the Oct4 ΔPE::GFP transgene was similarly induced in each of these lines by doxycycline (Figure 3D). Furthermore, induction of either ESRRB or NANOG in *Otx2*$^{+/-}$ cells increased expression of *Blimp1*, *Prdm14*, and *Ap2γ* mRNAs at day 6 (Figure 3E). This contrasts with induction of ESRRB in *Otx2*$^{+/+}$ cells, which did not increase *Blimp1* and *Prdm14* mRNA levels (Figure 1E). Interestingly, at the earlier time point of day 2 of

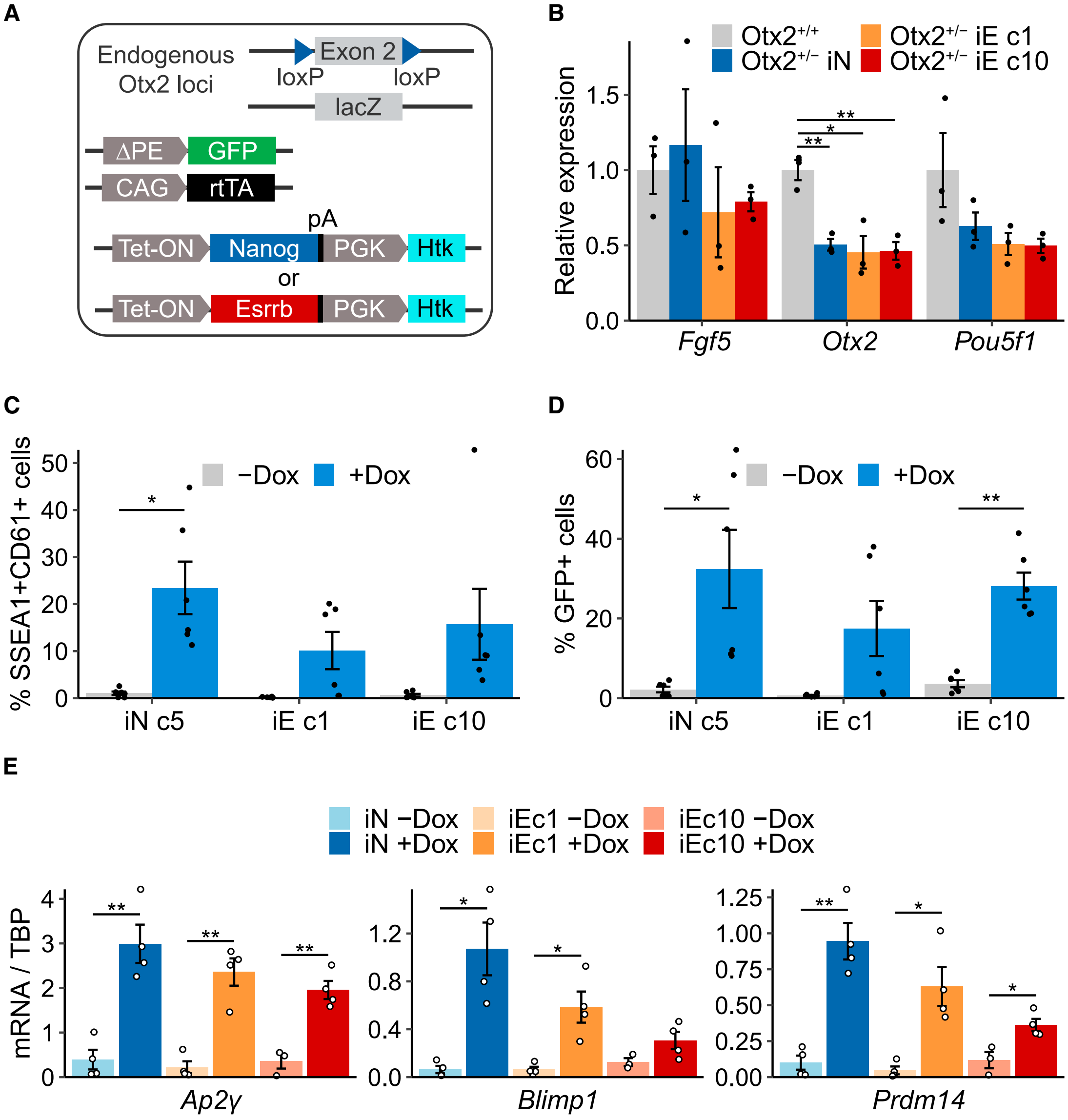

Figure 3. A reduced *Otx2* gene dose facilitates cytokine-free germline entry by ESRRB induction

(A) Diagram of *Otx2*$^{+/-}$: *Oct4* ΔPE-GFP ESCs carrying tetracycline-inducible Nanog (iN) or Esrrb (iE) transgenes.

(B) Relative expression levels of indicated mRNAs in *Otx2*$^{+/-}$ iN and iE (c1 and c10) EpiLCs in the absence of doxycycline. *Tbp*-normalized expression values are shown relative to the mean of *Tbp*-normalized expression of *Otx2*$^{+/+}$ samples. Bars are mean ± SEM and points are individual data measurements (n = 3 independent experiments).

(C) Size of PGCLC population (represented by SSEA1^{+}CD61^{+} cells) in the presence (+dox) or absence (−dox) of doxycycline at day 6 of cytokine-free PGCLC differentiation of the indicated *Otx2*$^{+/-}$ EpiLCs. Bars are mean ± SEM, and points are individual data measurements (n = 6 independent experiments).

(D) Proportion of *Oct4* ΔPE-GFP^{+} cells in the indicated *Otx2*$^{+/-}$ aggregates at day 6 of the cytokine-free PGCLC differentiation. Bars represent mean ± SEM of percentage of GFP+ cells, n = 6 independent experiments; points are individual data measurements.

(E) qRT-PCR analysis of the indicated mRNAs relative to *Tbp* expression in day 6 aggregates from (C).

Bars are mean ± SEM and points are individual data measurements (n = 4 independent experiments). *p < 0.05 and **p < 0.01 (t test).

differentiation, ESRRB induced lower levels of *Blimp1*, *Prdm14*, and *Ap2γ* mRNA expression in *Otx2*$^{+/-}$ cells than were achieved by NANOG induction (Figure S3A). This may indicate that ESRRB-induced germline entry in *Otx2*$^{+/-}$ cells is delayed compared with that induced by NANOG.

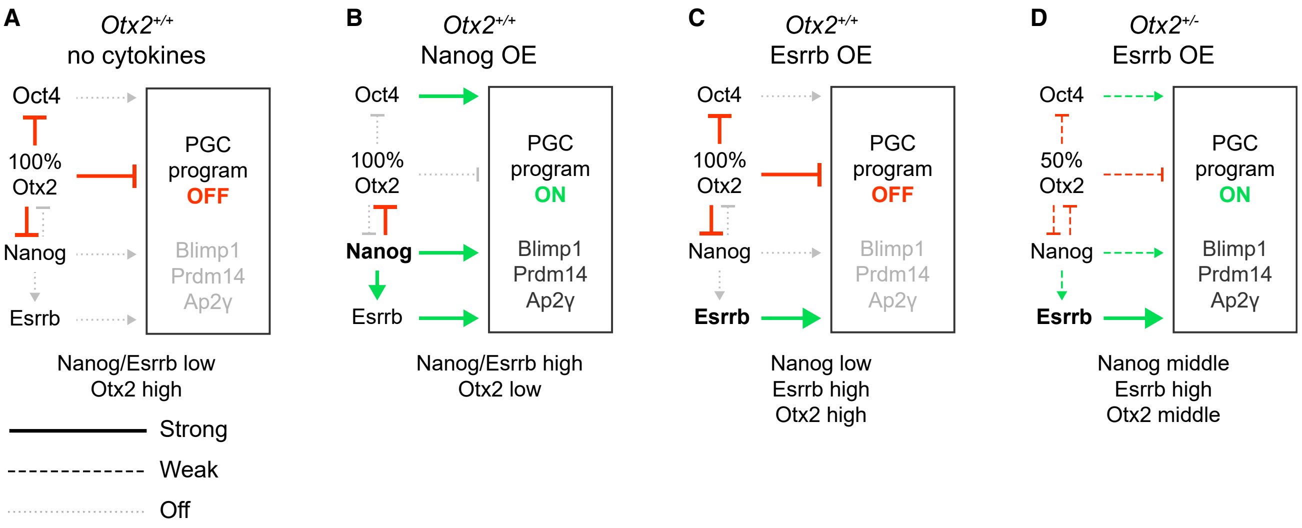

Figure 4. Proposed model of PGC induction

(A) In wild-type EpiLCs, in the absence of BMP and associated cytokines, OTX2 levels remain above the threshold level required to block PGCLC differentiation during the period of germline competence.

(B) If NANOG is overexpressed (OE) in EpiLCs at the start of differentiation, *Otx2* is repressed, leading to a decreased OTX2 level that no longer represses expression of the OTX2 target genes *Nanog* and *Oct4* (Di Giovannantonio et al., 2021). This increases the expression of OCT4, which together with NANOG and its downstream target ESRRB, provide positive regulatory inputs into the PGC GRN (centered on the TFs Blimp1, Prdm14, and Ap2γ), sufficient to drive PGCLC differentiation in the absence of cytokines.

(C) If ESRRB is overexpressed, there is no repression of *Otx2*, and the OTX2-mediated repression of *Nanog* and *Oct4* remains in place, blocking PGCLC differentiation.

(D) In *Otx2*-heterozygous EpiLCs, repression of *Nanog* and *Oct4* by OTX2 is diminished in strength. This enables induction of ESRRB to provide sufficient input into the PGC GRN (alongside mild inputs from NANOG and OCT4) to direct PGCLC differentiation.

DISCUSSION

A key function of PGC-specifying cytokines during the early stages of EpiLC-PGCLC differentiation is to rapidly downregulate *Otx2* (Zhang et al., 2018a). The results reported here show that NANOG induction in EpiLCs downregulates *Otx2* early during differentiation without requiring PGC-promoting cytokines. Importantly, these cytokines are also unnecessary for enforced NANOG expression in EpiLCs to induce PGCLCs (Murakami et al., 2016). The importance of the NANOG-mediated repression of *Otx2* for PGCLC differentiation under these conditions is shown by the reduction in the size of the PGCLC population when NANOG and OTX2 are simultaneously induced. Therefore, OTX2 depletion is essential for PGCLC specification by NANOG in the absence of cytokines.

ESRRB can substitute for NANOG in LIF-independent maintenance of mouse ESCs and in reprogramming of cells to naive pluripotency (Festuccia et al., 2012). In addition, knockin of *Esrrb* to *Nanog* rescues the reduced PGC numbers seen when *Nanog* is specifically deleted from the germ cell lineage (Zhang et al., 2018b). Furthermore, the failure of *Nanog*$^{-/-}$ cells to maintain a PGCLC population in the presence of PGC-promoting cytokines is rescued by enforced ESRRB expression (Zhang et al., 2018b). It was therefore surprising to see that ESRRB, unlike NANOG, cannot efficiently induce PGC transcription factors *Blimp1* and *Prdm14* in the absence of cytokines. However, ESRRB overexpression in *Nanog*$^{-/-}$ ESCs does not fully recapitulate the effect of NANOG overexpression on ESC self-renewal (Festuccia et al., 2012). Although NANOG and ESRRB share common target genes (Festuccia et al., 2012), distinct subsets of genes are regulated by either TF in ESCs (Sevilla et al., 2021). In addition, although the interactomes of NANOG and ESRRB contain common binding partners (Gagliardi et al., 2013; van den Berg et al., 2010), ESRRB, but not NANOG, interacts with Mediator, suggesting that ESRRB may function prominently in transcriptional initiation. Although both interactomes link to NuRD and PcG, NANOG also links to Sin3a and NcoR complexes. Therefore, NANOG may link more to transcriptional repression than ESRRB. These distinctions are consistent with the prominent role proposed for transcriptional repression during PGCLC differentiation (Kurimoto et al., 2008). Consistent with this, we show here that the failure of enforced ESRRB expression to recapitulate the PGCLC differentiation induced by ectopic NANOG may be due to the inability of ESRRB to rapidly downregulate *Otx2* within the

competence time window required to initiate PGC differentiation. When we tested the effect of ESRRB induction in *Otx2*$^{+/-}$ EpiLCs, ESRRB successfully activated expression of *Blimp1*, *Prdm14*, *Ap2γ*, and *Oct4* distal enhancer activity and surface expression of CD61/SSEA1. Therefore, the inability of ESRRB to induce the germline program is overcome by reducing the OTX2 level in the starting population.

In wild-type EpiLCs, PGC-promoting cytokines suppress *Otx2* expression and activate the PGC-specific gene regulatory network (GRN) (Zhang et al., 2018a). Our present findings, conducted in the absence of PGC-promoting cytokines, can be incorporated into current thinking about EpiLC-PGCLC differentiation as shown (Figure 4). In the absence of cytokines, OTX2 protein levels are sufficient to block activation of the PGCLC program. Recent findings indicate that this effect of OTX2 on the PCG program results largely from direct repression of *Nanog* and *Oct4* (Figure 4A) (Di Giovannantonio et al., 2021). Induction of transgenic Nanog in EpiLCs shifts the balance between the mutual antagonists NANOG and OTX2 in favor of NANOG (Figure 4B). Once OTX2 levels are sufficiently decreased, this enables NANOG to act on regulatory elements controlling genes in the PGC-specific GRN, including *Blimp1*, *Prdm14*, and *Ap2γ*, as previously suggested (Murakami et al., 2016). In addition, NANOG can activate *Esrrb*, which may also positively affect the PGC-specific GRN. Moreover, a reduction in OTX2 eliminates suppression of *Oct4*, which can also positively feed into the PGC-specific GRN (Figure 4B). If instead transgenic Esrrb is induced, a positive effect on the PGC-specific GRN can also be envisaged (Figure 4C). However, in this case, *Otx2* is not suppressed, OTX2 remains high, suppression of *Nanog* and *Oct4* is maintained, and the positive input into the PGC-specific GRN is insufficient to activate PGCLC differentiation. In contrast, in *Otx2*$^{+/-}$ cells, the balance between the mutual antagonists OTX2 and NANOG shifts (Figure 4D). In these conditions, induction of ESRRB may provide a sufficient positive effect on the PGC-specific GRN to direct some PGC differentiation due to a weakened suppression of *Nanog* and *Oct4* by OTX2. Further work will be required to bring greater clarity to the early events involved in germline specification prior to activation of the PGC-specific GRN.

EXPERIMENTAL PROCEDURES

PGCLC differentiation

Cytokine-free PGCLC differentiation with or without 1 μg/mL doxycycline was performed as previously described (Zhang et al., 2018a).

Additional methods can be found in the Supplemental experimental procedures.

Data and code availability

Code and data to reproduce the analysis and figures are available at https://github.com/MatusV8/Otx2het.

SUPPLEMENTAL INFORMATION

Supplemental information can be found online at https://doi.org/10.1016/j.stemcr.2021.11.013.

AUTHOR CONTRIBUTIONS

Conceptualization, M.V., J.Z., M.Z., and I.C.; Methodology, M.V., J.Z., and M.Z.; Investigation, M.V., J.Z., J.S., and M.Z.; Formal Analysis, M.V. and M.Z.; Writing – Original Draft, M.V., M.Z., and I.C.; Writing – Review & Editing, M.V., M.Z., and I.C.; Visualization, M.V.; Supervision, I.C. and M.Z.; Resources, I.C.; Funding Acquisition, M.Z. and I.C.

CONFLICT OF INTERESTS

The authors declare no competing interests.

ACKNOWLEDGMENTS

We thank members of the Chambers lab for discussions. This work was funded by grants to I.C. from the Biotechnology and Biological Sciences Research Council (BB/L002736/1) and the Medical Research Council (MR/T003162/1) of the United Kingdom and to M.Z. from the National Natural Science Foundation of China (32070800). M.V. was supported by a University of Edinburgh Principal's Career Development Scholarship.

Received: July 31, 2021
Revised: November 24, 2021
Accepted: November 25, 2021
Published: December 30, 2021

REFERENCES

Acampora, D., Di Giovannantonio, L.G., Garofalo, A., Nigro, V., Omodei, D., Lombardi, A., Zhang, J., Chambers, I., and Simeone, A. (2017). Functional antagonism between OTX2 and NANOG specifies a spectrum of heterogeneous identities in embryonic stem cells. Stem Cell Reports *9*, 1642–1659. https://doi.org/10.1016/j.stemcr.2017.09.019.

Acampora, D., Giovannantonio, L.G.D., and Simeone, A. (2013). Otx2 is an intrinsic determinant of the embryonic stem cell state and is required for transition to a stable epiblast stem cell condition. Development *140*, 43–55. https://doi.org/10.1242/dev.085290.

Carter, A.C., Davis-Dusenbery, B.N., Koszka, K., Ichida, J.K., and Eggan, K. (2014). Nanog-independent reprogramming to iPSCs with canonical factors. Stem Cell Reports *2*, 119–126. https://doi.org/10.1016/j.stemcr.2013.12.010.

Chambers, I., Silva, J., Colby, D., Nichols, J., Nijmeijer, B., Robertson, M., Vrana, J., Jones, K., Grotewold, L., and Smith, A. (2007). Nanog safeguards pluripotency and mediates germline development. Nature *450*, 1230–1234. https://doi.org/10.1038/nature06403.

Di Giovannantonio, L.G., Acampora, D., Omodei, D., Nigro, V., Barba, P., Barbieri, E., Chambers, I., and Simeone, A. (2021). Direct repression of Nanog and Oct4 by OTX2 modulates contribution of epiblast-derived cells to germline and somatic lineage. Development https://doi.org/10.1242/dev.199166.

Festuccia, N., Osorno, R., Halbritter, F., Karwacki-Neisius, V., Navarro, P., Colby, D., Wong, F., Yates, A., Tomlinson, S.R., and Chambers, I. (2012). Esrrb is a direct Nanog target gene that can substitute for Nanog function in pluripotent cells. Cell Stem Cell *11*, 477–490. https://doi.org/10.1016/j.stem.2012.08.002.

Gagliardi, A., Mullin, N.P., Ying Tan, Z., Colby, D., Kousa, A.I., Halbritter, F., Weiss, J.T., Felker, A., Bezstarosti, K., Favaro, R., et al. (2013). A direct physical interaction between Nanog and Sox2 regulates embryonic stem cell self-renewal. EMBO J. *32*, 2231–2247. https://doi.org/10.1038/emboj.2013.161.

Hayashi, K., de Sousa Lopes, S.M.C., and Surani, M.A. (2007). Germ cell specification in mice. Science *316*, 394–396. https://doi.org/10.1126/science.1137545.

Hayashi, K., Ohta, H., Kurimoto, K., Aramaki, S., and Saitou, M. (2011). Reconstitution of the mouse germ cell specification pathway in culture by pluripotent stem cells. Cell *146*, 519–532. https://doi.org/10.1016/j.cell.2011.06.052.

Hayashi, K., and Saitou, M. (2013). Generation of eggs from mouse embryonic stem cells and induced pluripotent stem cells. Nat. Protoc. *8*, 1513–1524. https://doi.org/10.1038/nprot.2013.090.

Heurtier, V., Owens, N., Gonzalez, I., Mueller, F., Proux, C., Mornico, D., Clerc, P., Dubois, A., and Navarro, P. (2019). The molecular logic of Nanog-induced self-renewal in mouse embryonic stem cells. Nat. Commun. *10*, 1–15. https://doi.org/10.1038/s41467-019-09041-z.

Kurimoto, K., Yabuta, Y., Ohinata, Y., Shigeta, M., Yamanaka, K., and Saitou, M. (2008). Complex genome-wide transcription dynamics orchestrated by Blimp1 for the specification of the germ cell lineage in mice. Genes Dev. *22*, 1617–1635. https://doi.org/10.1101/gad.1649908.

Lawson, K.A., Dunn, N.R., Roelen, B.a.J., Zeinstra, L.M., Davis, A.M., Wright, C.V.E., Korving, J., and Hogan, B.L.M. (1999). Bmp4 is required for the generation of primordial germ cells in the mouse embryo. Genes Dev. *13*, 424–436. https://doi.org/10.1101/gad.13.4.424.

Magnusdottir, E., Dietmann, S., Murakami, K., Guenesdogan, U., Tang, F., Bao, S., Diamanti, E., Lao, K., Gottgens, B., and Surani, M.A. (2013). A tripartite transcription factor network regulates primordial germ cell specification in mice. Nat. Cell Biol. *15*, 905–U322. https://doi.org/10.1038/ncb2798.

Mitsunaga, K., Araki, K., Mizusaki, H., Morohashi, K., Haruna, K., Nakagata, N., Giguère, V., Yamamura, K., and Abe, K. (2004). Loss of PGC-specific expression of the orphan nuclear receptor ERR-β results in reduction of germ cell number in mouse embryos. Mech. Dev. *121*, 237–246. https://doi.org/10.1016/j.mod.2004.01.006.

Murakami, K., Guenesdogan, U., Zylicz, J.J., Tang, W.W.C., Sengupta, R., Kobayashi, T., Kim, S., Butler, R., Dietmann, S., and Surani, M.A. (2016). NANOG alone induces germ cells in primed epiblast in vitro by activation of enhancers. Nature *529*, 403. https://doi.org/10.1038/nature16480.

Ohinata, Y., Payer, B., O'Carroll, D., Ancelin, K., Ono, Y., Sano, M., Barton, S.C., Obukhanych, T., Nussenzweig, M., Tarakhovsky, A., et al. (2005). Blimp1 is a critical determinant of the germ cell lineage in mice. Nature *436*, 207–213. https://doi.org/10.1038/nature03813.

Sevilla, A., Papatsenko, D., Mazloom, A.R., Xu, H., Vasileva, A., Unwin, R.D., LeRoy, G., Chen, E.Y., Garrett-Bakelman, F.E., Lee, D.-F., et al. (2021). An Esrrb and Nanog cell fate regulatory module controlled by feed forward loop interactions. Front. Cell Dev. Biol. *9*. https://doi.org/10.3389/fcell.2021.630067.

Urlinger, S., Baron, U., Thellmann, M., Hasan, M.T., Bujard, H., and Hillen, W. (2000). Exploring the sequence space for tetracycline-dependent transcriptional activators: novel mutations yield expanded range and sensitivity. Proc. Natl. Acad. Sci. U S A *97*, 7963–7968.

van den Berg, D.L.C., Snoek, T., Mullin, N.P., Yates, A., Bezstarosti, K., Demmers, J., Chambers, I., and Poot, R.A. (2010). An Oct4-centered protein interaction network in embryonic stem cells. Cell Stem Cell *6*, 369–381. https://doi.org/10.1016/j.stem.2010.02.014.

Vincent, S.D., Dunn, N.R., Sciammas, R., Shapiro-Shalef, M., Davis, M.M., Calame, K., Bikoff, E.K., and Robertson, E.J. (2005). The zinc finger transcriptional repressor Blimp1/Prdm1 is dispensable for early axis formation but is required for specification of primordial germ cells in the mouse. Development *132*, 1315–1325. https://doi.org/10.1242/dev.01711.

Weber, S., Eckert, D., Nettersheim, D., Gillis, A.J.M., Schäfer, S., Kuckenberg, P., Ehlermann, J., Werling, U., Biermann, K., Looijenga, L.H.J., et al. (2010). Critical function of AP-2 gamma/TCFAP2C in mouse embryonic germ cell maintenance. Biol. Reprod. *82*, 214–223. https://doi.org/10.1095/biolreprod.109.078717.

Yamaguchi, S., Kurimoto, K., Yabuta, Y., Sasaki, H., Nakatsuji, N., Saitou, M., and Tada, T. (2009). Conditional knockdown of Nanog induces apoptotic cell death in mouse migrating primordial germ cells. Development *136*, 4011–4020. https://doi.org/10.1242/dev.041160.

Yamaji, M., Seki, Y., Kurimoto, K., Yabuta, Y., Yuasa, M., Shigeta, M., Yamanaka, K., Ohinata, Y., and Saitou, M. (2008). Critical function of Prdm14 for the establishment of the germ cell lineage in mice. Nat. Genet. *40*, 1016–1022. https://doi.org/10.1038/ng.186.

Zhang, M., and Chambers, I. (2019). Segregation of the mouse germline and soma. Cell Cycle *18*, 3064–3071. https://doi.org/10.1080/15384101.2019.1672466.

Zhang, J., Zhang, M., Acampora, D., Vojtek, M., Yuan, D., Simeone, A., and Chambers, I. (2018a). OTX2 restricts entry to the mouse germline. Nature *562*, 595–599. https://doi.org/10.1038/s41586-018-0581-5.

Zhang, M., Leitch, H.G., Tang, W.W.C., Festuccia, N., Hall-Ponsele, E., Nichols, J., Surani, M.A., Smith, A., and Chambers, I. (2018b). Esrrb complementation rescues development of Nanog-null germ cells. Cell Rep. *22*, 332–339. https://doi.org/10.1016/j.celrep.2017.12.060.

Stem Cell Reports, Volume *17*

Supplemental Information

Differential repression of *Otx2* underlies the capacity of NANOG and ESRRB to induce germline entry

Matúš Vojtek, Jingchao Zhang, Juanjuan Sun, Man Zhang, and Ian Chambers

Figure S1 (related to figure 1)

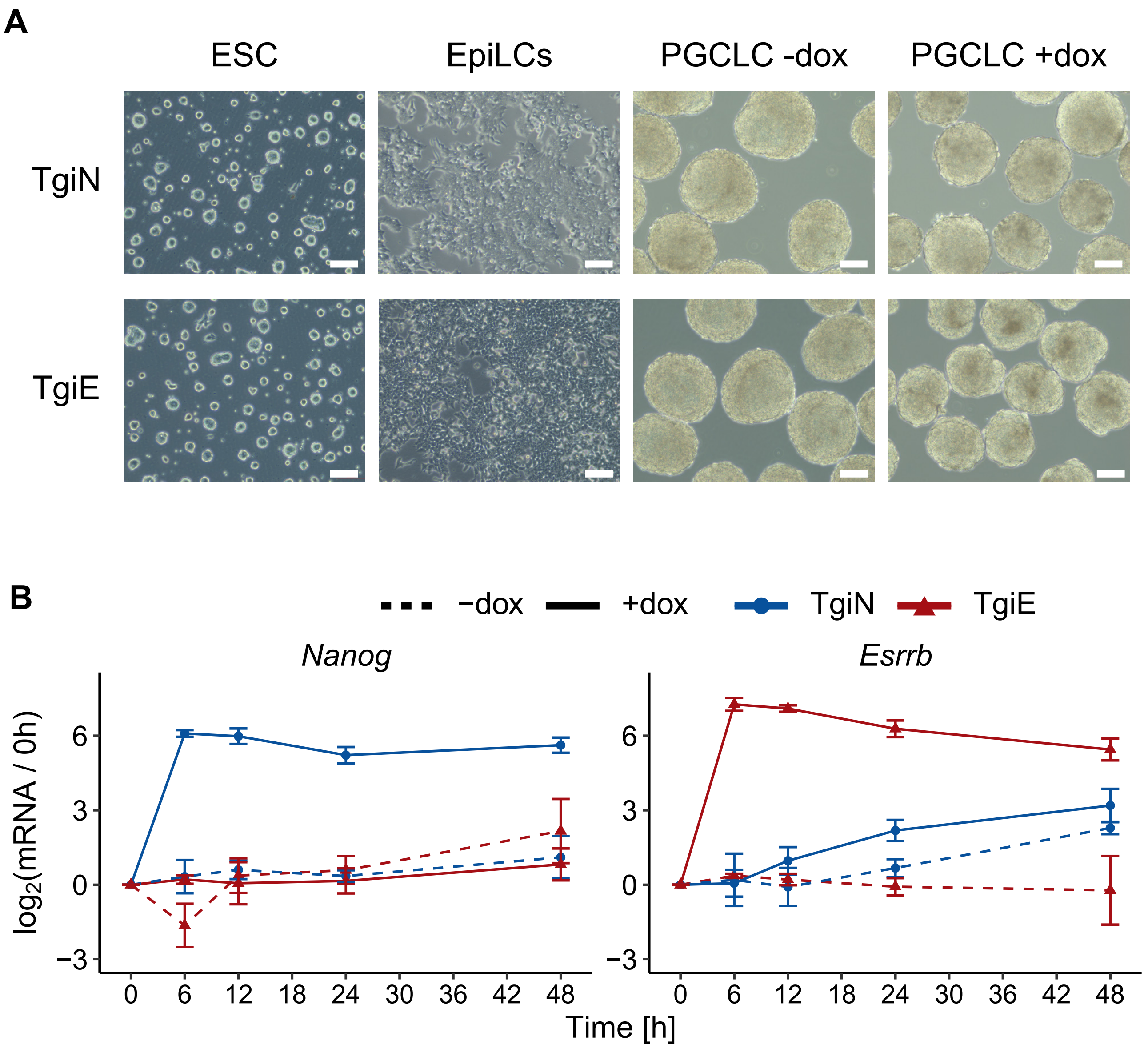

Figure S1: Validation of doxycycline inducible Nanog and Esrrb cell lines.
A) Photographs of TgiN and TgiE ESCs, EpiLCs and cell aggregates at day 6 of cytokine-free PGCLC differentiation in the presence (+dox) or absence (-dox) of doxycycline. White bars represent 100 µm.
B) Relative changes of *Esrrb* and *Nanog* mRNAs after aggregation of E14Tg2a TetON-Nanog (TgiN) and E14Tg2a TetON-Esrrb (TgiE) EpiLCs cultured in the absence (-dox) or presence (+dox) of doxycycline at the indicated time point. Lines, points and triangles represent mean log_2 fold-change (FC) differences between data points and the zero-time timepoint (mean ± SD, n = 3 independent experiments).

Figure S2 (related to figure 2)

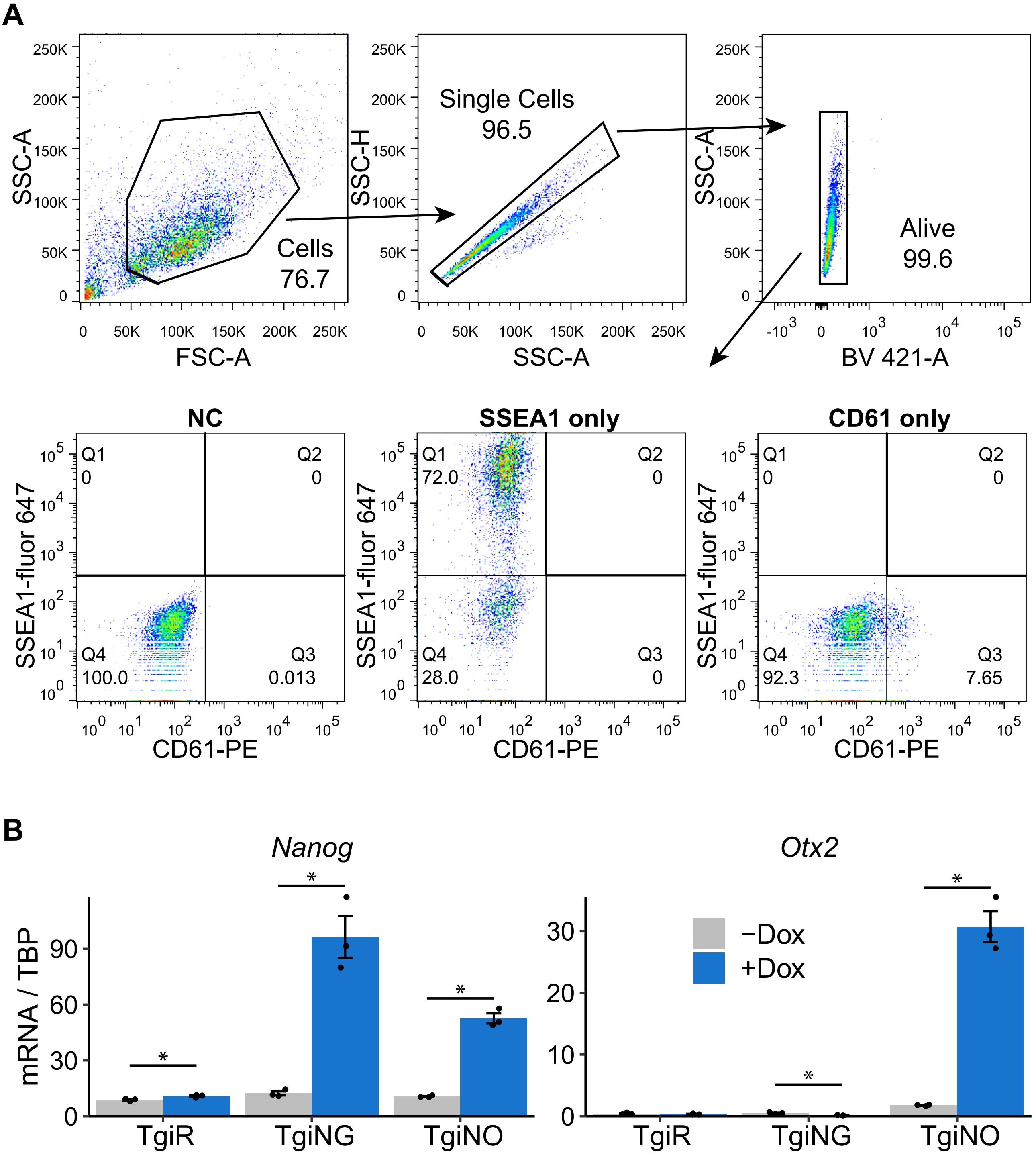

Figure S2: Gating strategy and activity of transgenes in TgiNG and TgiNO ESCs.
A) Gating strategy to quantify SSEA1+CD61+ cells. Single and living cells were assessed for fluor 647 and PE signal. Unstained negative control (NC), and single stained controls were used to draw gates. **B)** RT-qPCR quantification of *Nanog* and *Otx2* mRNAs in TgiR, TgiNG and TgiNO ESCs (related to Figure 1A) before and after 48 hours of doxycycline treatment. Bars are mean ± SEM, points are individual data measurements (n = 3 independent experiments). * $p < 0.05$ (t-test).

Figure S3 (related to figure 3)

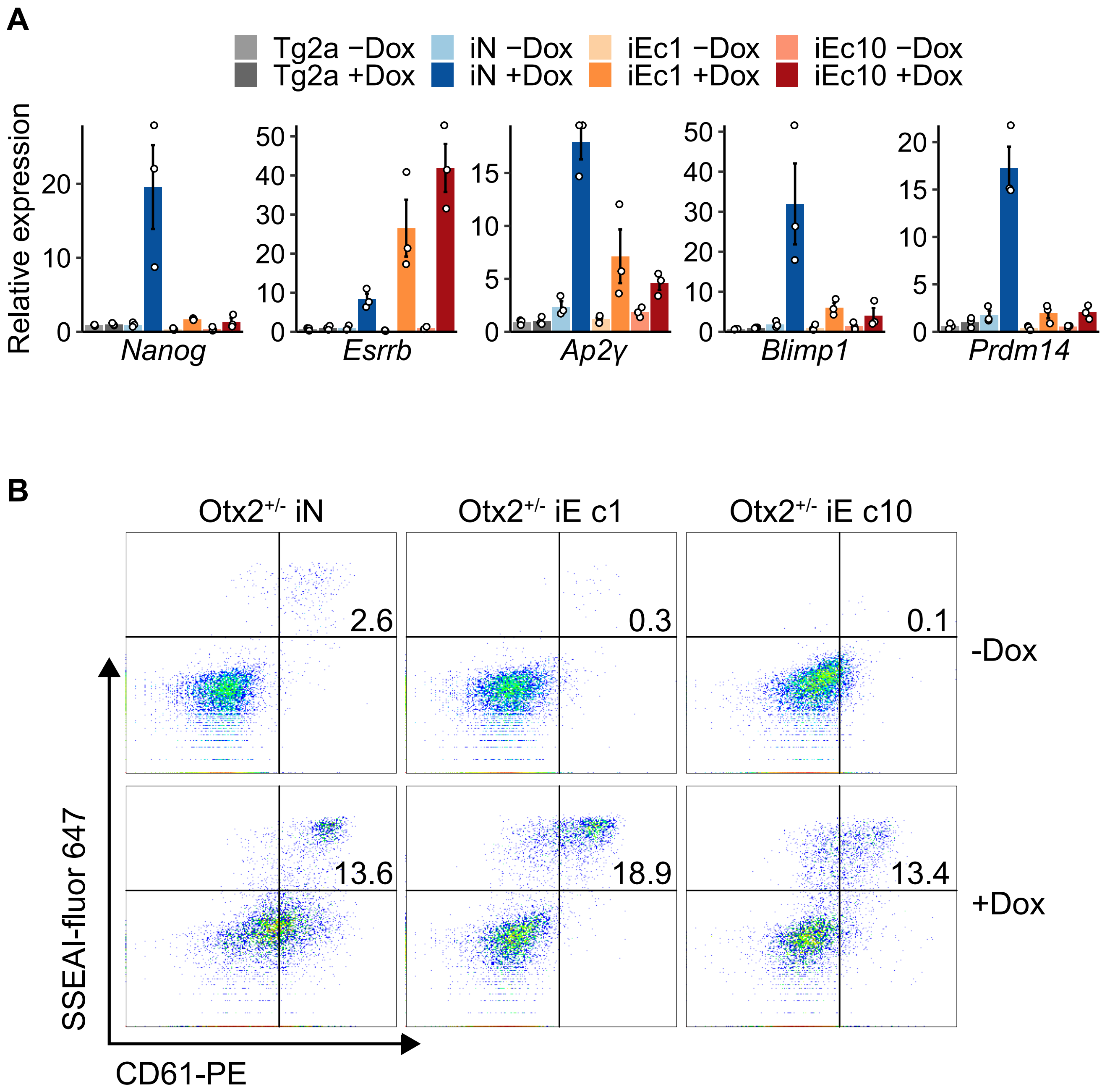

Figure S3: Cytokine-free differentiation of Nanog and Esrrb inducible *Otx2*$^{+/-}$ cell lines.
A) qRT-PCR analysis of the indicated mRNAs at day 2 of the cytokine-free PGCLC differentiation in the presence (+Dox) or absence (-Dox) of doxycycline. Bars are mean ± SEM, points are individual data measurements (n = 3 independent experiments) of relative expression normalised to the mean expression in Tg2a -Dox sample for each mRNA. **B)** Representative flow cytometry analysis of SSEA1 and CD61 expression at day 6 of cytokine-free PGCLC differentiation of the indicated cell lines in the presence (+Dox) or absence (-Dox) of doxycycline. Percentages of SSEA1+CD61+ populations are indicated.

Table S1: Primers used for qRT-PCR

Name	Forward primer (5' -> 3')	Reverse primer (5' -> 3')
Tbp	GGGGAGCTGTGATGTGAAGT	CCAGGAAATAATTCTGGCTCA
Pou5f1	CCCCAATGCCGTGAAGTTG	TCAGCAGCTTGGCAAACTGTT
Nanog	AGGATGAAGTGCAAGCGGTG	TGCTGAGCCCTTCTGAATCAG
Esrrb	CGATTCATGAAATGCCTCAA	CCTCCTCGAACTCGGTCA
Fgf5	AAAACCTGGTGCACCCTAGA	CATCACATTCCCGAATTAAGC
Otx2	GACCCGGTACCCAGACATC	GCTCTTCGATTCTTAAACCATACC
Pou3f1	CCATCGAGGTGGGTGTCAAA	CCTCCTTCTCCAGTTGCAGG
Blimp1	CCCCTCATCGGTGAAGTCTA	TGGTGGAACTCCTCTCTGGA
Prdm14	TCAATTCACTCCCGAAGTACCA	CCGGGGATGGCAGAAGTAAA
Ap2γ	ATCCCTCACCTCTCCTCTCC	CCAGATGCGAGTAATGGTCGG

Supplemental experimental procedures

Cell culture

All ESCs were derived from E14Tg2a (Tg2a) mouse ESCs (1) and were routinely cultured in Glasgow Minimum Essential Medium (GMEM; Sigma, cat. n. G5154) supplemented with 10% FCS (Gibco cat. n. 10270-106), 100 U/ml LIF (homemade), Non-essential amino acids (Invitrogen, cat. n. 11140-036), 1 mM sodium pyruvate (Invitrogen, cat. n. 11360-039), 2 mM L-glutamine (Invitrogen, cat. n. 25030-024) and 50 nM 2-mercaptoethanol (Gibco, cat. n. 31350-010) at density of 3-10x10^5 cells per cm^2 in culture flasks coated with 0.1% gelatine as previously described (2). Presence of mycoplasma contamination was routinely tested by MycoAlert™ Mycoplasma Detection Kit (Lonza; cat. n. LT07-318).

Generation of doxycycline inducible cell lines

To generate *Otx2*$^{+/+}$ Nanog or Esrrb inducible cell lines (TgiN, TgiE), firstly CAG-rtTAms2 was integrated into the *Rosa26* locus of E14Tg2a ESCs as described previously (3). E14Tg2a-rtTAms2 cells were transfected with piggyBac-TetON-Nanog-PGK-Hph-pA or piggyBac-TetON-Esrrb-PGK-Hph-pA together with pCMV-hyPBASE (4) plasmids using lipofectamine 3000 (Thermofisher; cat. n. L3000015).

To generate TgiNG and TgiNO cells, E14Tg2a-rtTAms2 cells were transfected with piggyBac-TetO-FRT5-TdTomato-2a-HygR-FRT3-tk (5) and selected for clones with robust TdTomato expression in presence of doxycycline and low levels of TdTomato in the absence of doxycycline. To integrate Nanog-T2A-GFP and Nanog-T2A-Otx2 cassettes, *Rosa26*: rtTAms2; E14Tg2a-TetON-TdTomato ESCs were transfected with pShuttle-TetON-FRT5-$(Flag)_3$-Nanog-T2A-GFP-IRES-PURO-FRT3 or pShuttle-TetON-FRT5-$(Flag)_3$-Nanog-T2A-Otx2-IRES-PURO-FRT3 together with pPGK-FLPobpa (Adgene #13793) using Lipofectamine 3000 (Thermofisher; cat. n. L3000015). Cells resistant to puromycin and without TdTomato expression in presence of doxycycline were selected.

To generate *Otx2*$^{+/-}$ ΔPE::GFP ESCs with doxycycline inducible transgenes, *Otx2*$^{lacZ/fl}$ ΔPE::GFP c11 ESCs (6) were transfected with piggyBac-TetON-Nanog-PGK-Hph-pA or piggyBac-TetON-Esrrb-PGK-Hph-pA together with pCMV-hyPBASE (4) and pCAG-rTtAsm2-IRES-BSD using lipofectamine 3000 (Thermofisher; cat. n. L3000015). Cells were selected with hygromycin and blasticidin for twelve days and single clones were picked. Two clones for Esrrb and one for Nanog were selected with capacity to induce the transgene and which express similar levels of *Otx2* to the parental cell line.

Flow cytometry

Cell aggregates were collected, washed with PBS and dissociated in 0.05 % Trypsin for ~10 minutes at 37 °C. Trypsin was neutralised with MEF medium [Glasgow Minimum Essential Medium (GMEM; Sigma, cat. n. G5154) supplemented with 10% FCS (Gibco cat. n. 10270-106), Non-essential amino acids (Invitrogen, cat. n. 11140-036), 1 mM sodium pyruvate (Invitrogen, cat. n. 11360-039), 2 mM L-glutamine (Invitrogen, cat. n. 25030-024) and 50 nM 2-mercaptoethanol (Gibco, cat. n. 31350-010)] and cells were passed through a cell strainer. Samples (~15%) from each cell suspension were combined in a separate tube for control samples. Cells were collected by centrifugation (3 min at 300g) and resuspended in 100 µl of MEF medium supplemented with 1:200 v/v Alexa Fluor 647 anti-mouse/human CD15 (SSEA-1) (Biolegend, cat. n. 125608) and 1:500 v/v PE anti-mouse/rat CD61 (Biolegend, cat. n. 104307). The control cells were resuspended in 300 µl of MEF medium and cell suspension was divided into three 100 µl parts. SSEA-I and CD61 antibodies were added to one fraction resulting in two single-stained control samples and one unstained control. Cells were incubated for 15 min at room temperature and washed twice with PBS before analysis at BD Fortessa 5 laser system. Flow cytometry data were analysed using FlowJo X 0.7 software. Live cells were gated based on DAPI signal, SSEA-I+CD61+ populations were gated based on the negative and single stain controls (see Figure S2).

qRT-PCR

Single cell suspension was collected by centrifugation (3 min at 300 g) and total RNA was extracted by Illustra™ RNAspin RNA Isolation Kit (GE Healthcare, cat. n. GE25-0500-72) according to manufacturer instructions. First strand cDNA was synthetised using 200-1000 ng RNA, SuperScript III reverse transcriptase (Thermofisher, cat. n. 18080044) and random hexamers at the final concentration 2.5 mM. cDNA was diluted 1:10 in nuclease-free grade water. qPCR mix was prepared by mixing 5 µl cDNA, 4.5 µl of 2x Takyon SYBR Green master mix (Eurogentec, cat. n. UF-NSMT-B0701) and 0.5 µl of 10 mM mixture of a primer pair (Table S1). Specificity and efficiency of used primer pairs were tested prior the experiments. Quantitative polymerase reactions were performed in two technical replicates for each independent replicate using LightCycler 450 instrument (Roche) with program: 3 min at 95 ºC, 45x (10s at 95 ºC, 20s at 60 ºC, 30s at 72 ºC). The cycle corresponding to the crossing point (Cp) was determined using the second derivative method and values for technical replicates were

averaged. Relative mRNA levels for each sample were determined by $2^{-\Delta\Delta Cp}$ method (7) using *Tbp* as a reference gene. For figures 1F, 2B and S1B, relative mRNA expression was normalised by calculating log2 of fold change between each time point and the 0h time point for each replicate. For figures 3B and S3A, values were normalised by diving each value by the mean value of the reference samples (*Otx2*$^{+/+}$ and Tg2a -Dox respectively).

Statistical analysis and data visualisation

Statistical analysis and visualisation was done using R v4.0.3 (8) and tidyverse packages (9). The number of independent experiments performed on different days (n) underlying each plot is reported in the figure legends. Summary statistics was reported as mean value ± standard error of the mean (SEM) or standard deviation (SD). Two-tailed t-test was used to compare treatment samples to the reference sample. Benjamin-Hochberg method was used to adjust p-values to correct for multiple testing.

References

1. Hooper M, Hardy K, Handyside A, Hunter S, Monk M. HPRT-deficient (Lesch–Nyhan) mouse embryos derived from germline colonization by cultured cells. Nature. 1987 Mar;326(6110):292–5.

2. Smith AG. Culture and differentiation of embryonic stem cells. Journal of Tissue Culture Methods. 1991 Jun 1;13(2):89–94.

3. Bressan RB, Dewari PS, Kalantzaki M, Gangoso E, Matjusaitis M, Garcia-Diaz C, et al. Efficient CRISPR/Cas9-assisted gene targeting enables rapid and precise genetic manipulation of mammalian neural stem cells. Development. 2017 Feb 15;144(4):635–48.

4. Yusa K, Zhou L, Li MA, Bradley A, Craig NL. A hyperactive piggyBac transposase for mammalian applications. Proc Natl Acad Sci U S A. 2011 Jan 25;108(4):1531–6.

5. Festuccia N, Osorno R, Halbritter F, Karwacki-Neisius V, Navarro P, Colby D, et al. Esrrb Is a Direct Nanog Target Gene that Can Substitute for Nanog Function in Pluripotent Cells. Cell Stem Cell. 2012 Oct;11(4):477–90.

6. Zhang J, Zhang M, Acampora D, Vojtek M, Yuan D, Simeone A, et al. OTX2 restricts entry to the mouse germline. Nature. 2018 Oct;562(7728):595–9.

7. Livak KJ, Schmittgen TD. Analysis of Relative Gene Expression Data Using Real-Time Quantitative PCR and the 2−ΔΔCT Method. Methods. 2001 Dec 1;25(4):402–8.

8. Team RC, others. R: A language and environment for statistical computing. 2013;

9. Wickham H, Averick M, Bryan J, Chang W, McGowan LD, François R, et al. Welcome to the Tidyverse. Journal of Open Source Software. 2019 Nov 21;4(43):1686.
